# Spatial Patterns of Brain Activity Preferentially Reflecting Transient Pain and Stimulus Intensity

**DOI:** 10.1093/cercor/bhz026

**Published:** 2019-03-07

**Authors:** M Liang, Q Su, A Mouraux, G D Iannetti

**Affiliations:** 1 School of Medical Imaging and Tianjin Key Laboratory of Functional Imaging, Tianjin Medical University, Tianjin, China; 2 Department of Neuroscience, Physiology and Pharmacology, University College London, London, UK; 3 Institute of Neuroscience (IoNS), Université catholique de Louvain, Brussels, Belgium; 4 Neuroscience and Behaviour Laboratory, Istituto Italiano di Tecnologia, Rome, Italy

**Keywords:** fMRI, multivariate pattern analysis, pain, pain matrix, saliency

## Abstract

How pain emerges in the human brain remains an unresolved question. Neuroimaging studies have suggested that several brain areas subserve pain perception because their activation correlates with perceived pain intensity. However, painful stimuli are often intense and highly salient; therefore, using both intensity- and saliency-matched control stimuli is crucial to isolate pain-selective brain responses. Here, we used these intensity/saliency-matched painful and non-painful stimuli to test whether pain-selective information can be isolated in the functional magnetic resonance imaging responses elicited by painful stimuli. Using two independent datasets, multivariate pattern analysis was able to isolate features distinguishing the responses triggered by (1) intensity/saliency-matched painful versus non-painful stimuli, and (2) high versus low-intensity/saliency stimuli regardless of whether they elicit pain. This indicates that neural activity in the so-called “pain matrix” is functionally heterogeneous, and part of it carries information related to both painfulness and intensity/saliency. The response features distinguishing these aspects are spatially distributed and cannot be ascribed to specific brain structures.

## Introduction

In the last two decades, hundreds of studies using functional magnetic resonance imaging (fMRI) have shown that transient noxious stimuli eliciting painful percepts generate consistent responses within an array of brain structures that have often been referred to as the “pain matrix” (Tracey and Mantyh 2007; Iannetti and Mouraux 2010, 2015; Garcia-Larrea and Peyron 2013). This array typically includes the thalamus, the primary and secondary somatosensory cortices, the insula and the anterior/mid cingulate cortex (Apkarian et al. 2005; Tracey and Mantyh 2007; Duerden and Albanese 2013; Iannetti et al. 2013). The functional significance of these brain responses has triggered heated debates in pain neuroscience (Legrain et al. 2011; Iannetti et al. 2013; Eisenberger 2015; Iannetti and Mouraux 2015; Hu and Iannetti 2016; Wager et al. 2016; Mouraux and Iannetti 2018). A number of researchers have concluded that these responses, or at least a subset of them, reflect pain-specific neural activity (e.g., Treede et al. 1999, Tracey and Mantyh 2007, Goksan et al. 2015, Lieberman and Eisenberger 2015), on the basis that (1) they are consistently observed during pain, and (2) their magnitude is often graded with the intensity of perceived pain. These arguments, largely based on reverse inference (for a review see Iannetti et al., 2013; Mouraux and Iannetti 2018), have prompted researchers to draw strong conclusions based on the observation of activation of this “pain matrix,” like inferring that patients in minimally conscious state are able to experience pain (Boly et al. 2008), or that social pain shares neural mechanisms with physical pain (Eisenberger et al. 2003; Macdonald and Leary 2005; Kross et al. 2011; Eisenberger 2015; Goksan et al. 2015). However, the notion that these responses represent a signature for pain has been questioned given that these brain areas also respond to noxious stimuli in pain free patients (Salomons et al. 2016), or to salient but non-painful tactile, auditory and visual stimuli in healthy subjects (Downar et al. 2002, 2003; Mouraux and Iannetti 2009; Mouraux et al. 2011), and that the correlation between the magnitude of activation and perceived pain intensity can be easily disrupted by manipulating the saliency of the eliciting stimulus (Treede et al. 2003; Clark et al. 2008; Iannetti et al. 2008). This has led to an alternative hypothesis—that these responses are largely unspecific for pain and, instead, predominantly reflect the activation of a *supramodal* cortical network, possibly related to detecting, orienting attention towards and reacting to the occurrence of significant events, regardless of the sensory modality through which these events are conveyed (Downar et al. 2000, 2001, 2002; Iannetti and Mouraux 2010, 2015; Legrain et al. 2011). This alternative hypothesis is also supported by evidence that the cingulate cortex and the anterior insula show fMRI responses in a wide number of tasks that do not entail nociceptive stimulation, like saliency processing (Menon and Uddin 2010), awareness (Craig 2009), negative affect, cognitive control (Isnard et al. 2011; Shackman et al. 2011; Lieberman and Eisenberger 2015; Corradi-Dell’Acqua et al. 2016), executive processing (Vogt et al. 1992), and conflict monitoring (Botvinick et al. 2004), although some recent studies suggested that more sophisticated analyses may reveal specific pain processing in some parts of cingulate cortex (Liang et al. 2013; Kragel et al. 2018) and anterior insula (Fazeli and Buchel 2018; Sharvit et al. 2018). Regardless of the actual function(s) of this network, these observations emphasize the necessity to control for both stimulus saliency and stimulus intensity when aiming to identify “pain-specific” neural activity. Here, we refer to stimulus saliency as the ability of the stimulus to transiently capture attention (Downar et al. 2002; Legrain et al. 2011). For example, a loud sound such as a sudden bang of a door closed by the wind can be as salient as or even more salient than a short-lasting and localized painful stimulus.

Given that the existence of neural activity specifically determining painful percepts is out of question, the problem is really about whether this activity is reflected and can be isolated from the fMRI signal (Mouraux and Iannetti 2018). In most fMRI studies, the brain responses elicited by painful stimuli have been identified using mass-univariate analyses that measure average regional activity. Exploiting the rapid advancement of multivariate pattern analysis (MVPA) of fMRI data, which offers the unprecedented ability to detect small differences in the spatial patterns of brain activation across experimental conditions, a few recent studies have attempted to identify responses reflecting brain activities that are unique for pain (Liang et al. 2013; Wager et al. 2013; Krishnan et al. 2016). For example, although the anterior cingulate cortex (ACC) and the anterior insula were found to be active in many painful and non-painful conditions (Shackman et al. 2011; Wager et al. 2016), the spatial patterns of neural activities within these regions were able to track somatic but not vicarious pain intensity (Krishnan et al. 2016); and the spatial patterns of neural activities within any primary sensory cortices were distinguishable between painful and non-painful conditions (Liang et al. 2013). Particularly, Wager et al. (2013) showed that the fMRI responses elicited by nociceptive stimuli could be used to predict successfully the intensity of pain across individuals. Based on this successful prediction, as well as on the observation that the same classifier was unable to predict social pain, they affirmed to have identified a “neurological pain signature” (NPS). Moreover, the NPS was found to be able to distinguish thermal pain from non-painful warmth, anticipated pain, pain recall (Wager et al. 2013), social rejection (Woo et al. 2014), aversive images (Chang et al. 2015), and observed pain (Krishnan et al. 2016), and it could be generalized to mechanical and electrical pain (Krishnan et al. 2016). However, in all these studies, the saliency and aversiveness of non-painful conditions was either not explicitly matched with the painful condition (e.g., warmth vs. pain), or the non-painful conditions were not in the somatic domain (e.g., social rejection or aversive images vs. pain). Therefore, as reasoned above, it remains unclear whether the pattern of fMRI activity allowing the prediction was specifically related to the painful quality of the percept elicited by the nociceptive stimuli, or whether the same pattern could be elicited by equally-salient, non-painful sensory stimuli (Hu and Iannetti 2016; Mouraux and Iannetti 2018).

Here, we asked whether the brain responses elicited by painful stimulation and measured using fMRI (often referred to as the “pain matrix”), contain information selective to pain after the crucial factors of stimulus intensity and saliency are controlled for. To this end, we performed MVPA on two independent datasets collected using different MRI scanners to test the presence of reproducible response patterns that can predict, across different participants, whether the fMRI responses are elicited (1) by a transient nociceptive stimulus perceived as painful versus a transient intensity- and saliency-matched (from hereinafter, “intensity/saliency-matched”) non-painful auditory, visual or tactile stimulus, as well as (2) by salient versus non-salient (or equally, high- vs. low-intensity) stimuli, regardless of whether they are perceived as painful. Note that here we did not intend to disentangle the effects of stimulus saliency and intensity: although saliency and intensity can be experimentally dissociated using specific paradigms (e.g., Iannetti et al. 2008), in most experimental paradigms, including those used to collect Datasets 1 and 2, they are tightly coupled (Supplementary Fig. S6a). The availability of datasets collected from different individuals in different scanners allowed us to test the reproducibility and generalizability of the identified response patterns. Here, reproducibility is referred to replicating results in two different datasets separately, and generalizability is referred to testing the pattern identified from one dataset on another dataset.

## Materials and Methods

Two independent datasets were used in the present study. To test whether pain-specific responses can be identified within the so-called fMRI “pain matrix,” we performed both univariate analyses and MVPA within-datasets and across-datasets, comparing the fMRI responses elicited by intensity/saliency-matched painful and non-painful stimuli. To additionally test whether fMRI responses related to stimulus intensity/saliency can be also identified within the so-called “pain matrix,” we further compared, using MVPA, both within- and across-datasets, the responses elicited by high-saliency vs. low-saliency stimuli (Dataset 1) and by high-intensity versus low-intensity stimuli (Dataset 2) regardless of their sensory modality. An overview of the analyses performed is provided in Supplementary Fig. S1.

### Participants, Sensory Stimuli, Experimental Design and Data Acquisition

#### Dataset 1

This dataset was collected at the University of Oxford, UK. Fourteen right-handed healthy participants (6 females, aged 20–36 years) took part in the study after providing written informed consent. The local Ethics Committee approved the experimental procedures. Supplementary Fig. S2a illustrates the experimental design. In brief, participants received stimuli of four sensory modalities: pain (infrared laser pulses delivered to the foot dorsum), touch (transcutaneous electrical stimulation of the superficial peroneal nerve at the level of the ankle), vision (a bright white disk presented above the right foot) and audition (right-lateralised 800 Hz tones delivered through pneumatic earphones). The intensity of each of the four types of stimuli was adjusted individually for each participant to achieve similar stimulus saliency across the four sensory modalities (Renier et al. 2009; Spence 2009). Importantly, only laser stimuli were perceived as painful.

3 T fMRI data were acquired in a single session divided into four runs (41 contiguous 3.5-mm-thick axial slices; 3 × 3 mm in-plane resolution; repetition time of 3 s). Each run consisted of a stimulation period of 32 stimuli (8 stimuli/modality) pseudo-randomly delivered (inter-stimulus interval 10–19 s, <3 consecutive stimuli of the same modality), followed by a rating period of ~2 min during which participants rated the saliency of each stimulus type using a visual scale. At the end of the experiment, a T1-weighted structural image (1-mm-thick axial slices; 1 × 1 mm in-plane resolution) was acquired for spatial registration. This dataset has been analysed previously, and detailed information about experimental design and data acquisition can be found in (Mouraux et al. 2011; Liang et al. 2013).

#### Dataset 2

This dataset was collected at Tianjin Medical University, China. Fifty-one right-handed healthy participants (34 females, aged 18–29 years) took part in the study after providing written informed consent. The local Ethics Committee approved the experimental procedures. Supplementary Fig. S2b illustrates the experimental design. Participants received stimuli of two sensory modalities: pain (infrared laser pulses delivered to the foot dorsum) and touch (transcutaneous electrical stimulation of the superficial peroneal nerve at the level of the ankle). The stimulation devices were identical in Datasets 1 and 2. For each modality, two stimulus intensity levels (low and high) were used. The actual intensity of each level for each modality was adjusted individually for each participant before the fMRI experiment: the participant rated 3 and 6 (on a 0–10 scale) for the low and high intensities, respectively, for each modality.

3T fMRI data were acquired using a simultaneous multi-slices, gradient echo, echo-planar imaging sequence with the following parameters: echo time (TE) = 30 ms, repetition time (TR) = 800 ms, field of view (FOV) = 222 × 222 mm, matrix = 74 × 74, in-plane resolution = 3 × 3 mm, flip angle (FA) = 54°, slice thickness = 3 mm, no gap between slices, number of slices = 48, slice orientation = transversal, bandwidth = 1 690 Hz/Pixel, PAT (Parallel Acquisition Technique) mode, slice acceleration factor = 4, phase encoding acceleration factor = 2. The fMRI experiment included two sessions for each participant. Each session consisted of 3 “pain” blocks (in which only painful stimuli were delivered) and 3 “touch” blocks (in which only tactile stimuli were delivered). Each block consisted of four trials of the same modality. In each trial, a fixation cross was presented at the center of a screen during the first 15 s, and a single stimulus was delivered at a random onset between 2 s and 12 s. After a variable interval (3–13 s) following the stimulus, participants were instructed to rate the perceived intensity using a visual analog scale (0–10) presented on the screen for 10 s, by moving one of the two buttons with their right index or middle finger, resulting in a total duration of 25 s for each trial. Therefore, each session lasted 10 min during which 12 painful stimuli and 12 tactile stimuli were delivered. A T1-weighted structural image (two inversion contrast magnetization prepared rapid gradient echo sequence, MP2RAGE) was acquired using the following parameters: TR = 4 000 ms, TE = 3.41 ms, inversion times (TI1/TI2) = 700 ms/2110 ms, FA1 = 4°, FA2 = 5°, matrix = 256 × 240, FOV = 256 × 240 mm, number of slices = 192, in-plane resolution = 1 × 1 mm, slice thickness = 1 mm, slice orientation = sagittal.

### Regions of Interest Selection

**Figure 1.**
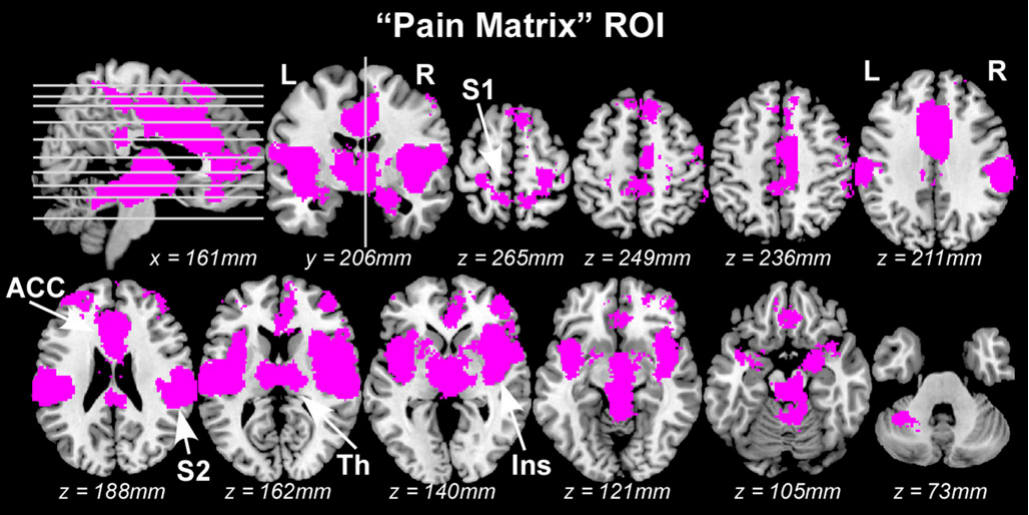
The “pain matrix” areas used in the mass-univariate GLM analyses and in the MVPA. L: left; R: right; S1: primary somatosensory cortex; S2: secondary somatosensory cortex; Ins: insula; ACC: anterior cingulate cortex; Th: thalamus.

Regions of interest (ROIs) defining the “pain matrix” were generated by merging three different masks. Mask 1, labeled “Neurosynth pain,” was created using exactly the same procedure described by Wager et al. (2013). Briefly, a mask composed of brain areas commonly activated by painful stimulation was created *a priori* using the automated meta-analysis toolbox Neurosynth (www.neurosynth.org) (Yarkoni et al. 2011). This mask was based on regions showing consistent results across 224 published studies frequently using the word “pain” (out of 4 393 total studies in the database) in a “reverse inference” analysis, which was an analysis of the 2 × 2 contingency table of counts of [activated vs. non-activated] × [pain vs. non-pain] voxels. Studies were counted as involving “pain” if they mentioned “pain” more than 1 time per 1 000 words in the study (the default value in neurosynth) and thresholded at *q* < 0.05 (corrected for False Discovery Rate; *P* < 0.0072). The mask included 30 432 voxels (2 × 2 × 2 mm) showing a positive blood-oxygen-level-dependent (BOLD) response to painful stimuli (these voxels were 8.64% of the total number of voxels composing the standard SPM8 brain mask [*brainmask.nii*]). Voxels showing a negative BOLD response to painful stimuli were not included in the mask. Importantly, this mask did not cover the S1 area corresponding to the representations of the leg and foot, as most studies included in the meta-analysis did not deliver somatosensory stimuli to the leg or foot. Given that stimuli used in the present study were delivered to the foot, two additional masks were generated. Mask 2, labeled “Neurosynth foot,” was created using the same procedure as in Mask 1, except that the keyword for the meta-analysis was “foot” instead of “pain.” The resulting mask included not only the S1 foot area, but also other areas in the brain. Therefore, Mask 3, labeled “Individual S1 foot,” was created based on each individual structural MRI using the following two steps. First, a region of interest circumscribing S1 was defined in the Montreal Neurological Institute (MNI) space using the Jülich probabilistic histological atlas (Eickhoff et al. 2005). Second, this ROI was transformed back to each individual space where it was trimmed to include only the mesial hemispheric wall (i.e., the putative foot representation area of S1), and then transformed again to standard MNI space (Penfield and Boldrey 1937), as described in a previous study (Mouraux et al. 2011). Finally, a group-level ROI was defined by taking the union of all normalized individual S1 foot ROIs. By taking the overlap between Mask 2 (“Neurosynth foot”) and Mask 3 (“Individual S1 foot”), we created a specific ROI defining the foot area of S1. A final “pain matrix” ROI was created by taking the union of Mask 1 and this S1 foot ROI, including 30 789 voxels in total (Fig. 1).

To test whether pain-specific information is present outside the “pain matrix,” we created two masks (“non-pain matrix” masks) using voxels not included in the “pain matrix” ROI. The first “non-pain matrix” mask, containing 208 580 voxels, was created by taking all voxels in the brain which were not included in the “pain matrix” mask (Supplementary Fig. S3a). As the number of voxels may affect the MVPA classification accuracy (Friston et al. 2003; Lorenz et al. 2003; Protzner and McIntosh 2006), we created the second “non-pain matrix” mask, containing the same number of voxels with the “pain matrix” mask (30 789 voxels), using the following steps: first, the initial “non-pain-matrix” mask was eroded by four layers using the software package FSL (www.fmrib.ox.ac.uk/fsl), resulting in a mask containing 41 329 voxels; second, as this mask is still considerably larger than the “pain matrix” mask, we randomly selected 30 789 voxels from the 41 329 voxels included in the eroded mask (Supplementary Fig. S4a).

These ROIs were resampled to 3 × 3 × 3 mm and used for the classification analyses conducted on Dataset 2, as well as for the across-datasets classifications. After resampling, the numbers of voxels in these masks were: 9 188 (“pain matrix” mask), 61 682 (the first “non-pain matrix” masks), and 9 064 (the second “non-pain matrix” mask).

### Saliency or Intensity Matching

#### Dataset 1

To rule out the possible confound of differences in stimulus saliency when comparing brain responses to painful and non-painful stimuli, we selected a sub-group of subjects to optimally match saliency differences across sensory modalities. For example, for the comparison of “pain vs. touch,” five subjects rated the saliency for pain lower than touch and nine subjects rated the saliency for pain higher than touch. Out of these nine subjects, we selected five subjects with a saliency difference matching the saliency difference of the five subjects who rated the saliency for pain lower than touch, to completely counter-balance the saliency difference between pain and touch across subjects (Table 1). Using the same procedure, we selected 10 subjects for the “pain vs. audition” and 8 subjects for the “pain vs. vision” comparison (Table 1). The same sub-groups of subjects were also used in the subsequent ROI-wise univariate analysis and MVPA. Using this approach, the subjective ratings of stimulus saliency were virtually identical between sensory modalities, in each of the three two-way classifications, confirmed by a two-tailed paired *t*-test as well as a model selection method based on Bayes Factor (Rouder et al. 2009; Morey et al. 2016; Dienes et al. 2018) (statistical values are detailed in Table 1).

**Table 1:** Differences in stimulus saliency and in head motion between two sensory modalities in each classification task of Dataset 1

	Pain vs. Touch	Pain vs. Audition	Pain vs. Vision
Saliency ratings			
Mean ± SD	Pain: 5.50 ± 2.30	Pain: 5.48 ± 2.24	Pain: 4.96 ± 2.13
	Touch: 5.53 ± 2.10	Audition: 5.41 ± 3.37	Vision: 4.91 ± 1.89
*t*/*P*-value	*t*_(9)_ =−0.09, *P* = 0.93	*t*_(9)_ = 0.05, *P* = 0.96	*t*_(7)_ = 0.04, *P* = 0.97
Bayes factor	BF_01_ = 4.29	BF_01_ = 4.30	BF_01_ = 3.91
Head motion (mm)			
Mean ± SD	Pain: 0.07 ± 0.07 mm	Pain: 0.06 ± 0.07 mm	Pain: 0.04 ± 0.01 mm
	Touch: 0.06 ± 0.04 mm	Audition: 0.08 ± 0.10 mm	Vision: 0.04 ± 0.02 mm
*t*/*P*-value	*t*_(9)_ = 0.88, *P* = 0.40	*t*_(9)_ =−1.49, *P* = 0.17	*t*_(7)_ = 0.004, *P* = 0.99
Bayes factor	BF_01_ = 3.02	BF_01_ = 1.68	BF_01_ = 3.92

#### Dataset 2

As participants were asked to rate the intensity of each stimulus in Experiment 2, a subset of stimuli was selected in each participant to match the perceived intensity of painful and tactile stimuli (pain: mean ± SD = 4.33 ± 1.04; touch: mean ± SD = 4.34 ± 1.03; paired *T*-test: *T* = −1.46, *P* = 0.15; Bayes factor BF_01_ = 3.27). The detailed procedure for matching stimulus intensity between modalities is as follows: for a laser stimulus with perceived intensity rating of *r*, all electrical stimuli with perceived intensity within the range of [*r*−0.5, *r* + 0.5] were identified, and the electrical stimulus with the closest rating was selected to pair with this particular laser stimulus. If no electrical stimulus was identified within this range, this laser stimulus was discarded from further analyses. In this way, the selected pairs of laser and electrical stimuli that were matched on a trial-by-trial level in terms of their perceived intensity (Supplementary Fig. S5). As stimulus saliency is closely related to stimulus intensity in this experimental setting, matching intensity between pain and touch was considered as similar to matching saliency in the present study.

#### Relationship between Intensity, Saliency and Valence

As we collected saliency ratings in Dataset 1 and intensity ratings in Dataset 2, we performed a third psychophysical experiment where we collected three types of ratings: stimulus intensity, stimulus saliency and stimulus valence. We were therefore able to test whether stimulus saliency or valence were similar for intensity-matched painful and tactile stimuli (details about experimental design and data analysis are provided in Supplementary Methods and Results). We found that both saliency and valence ratings were highly correlated with intensity ratings (Supplementary Fig. S6a&c): there was strong evidence for a positive correlation between saliency and intensity ratings (*R* = 0.86, *P* = 6.92 × 10^−14^), and for a negative correlation between valence and intensity ratings (R = −0.49, *P* = 7.66 × 10^−4^). Moreover, when comparing intensity-matched painful and tactile stimuli, both saliency and valence ratings were similar (Supplementary Fig. S6b&d): for saliency ratings, *T* = −0.0055, *P* = 0.9956 and the Bayes Factor (BF_01_) = 4.49; for valence ratings, *T* = −0.77, *P* = 0.44 and BF_01_ = 3.44.

### Mass-Univariate General Linear Modeling

To test whether differences in neural activity elicited by painful and non-painful stimuli can be detected using standard fMRI analysis, we performed a mass-univariate general linear modeling (GLM) analysis (Friston et al. 1995) for all voxels within the pre-defined “pain matrix” ROI.

#### Dataset 1

FMRI data were analysed using FSL (www.fmrib.ox.ac.uk/fsl). For each individual dataset, the first four volumes were discarded to allow for signal equilibration, and the remaining 736 volumes were motion corrected. The data was further spatially smoothed with a Gaussian kernel with a full-width at half-maximum (FWHM) of 8 mm and temporally high-pass filtered (1/100 Hz cutoff). For each participant, first-level statistical parametric maps were obtained using a GLM with regressors modeling the occurrence of each of the four types of stimuli (pain, touch, audition and vision), their corresponding temporal derivatives and additional regressors corresponding to the head motion parameters. Three contrast analyses were performed to identify voxels showing significant differences in the fMRI responses to painful and non-painful stimuli (i.e., “pain vs. touch,” “pain vs. audition” and “pain vs. vision”). The resulting contrast maps were further normalized to standard MNI space and re sampled to 2 × 2 × 2 mm voxel size. A second-level analysis (one-sample two-tailed *t*-test) was then performed using the first-level single-subject contrast maps to obtain the group-level statistical parametric maps for each comparison. Voxels showing significant differences between pain and other modalities were identified using a false discovery rate (FDR) of *q *< 0.05.

#### Dataset 2

Data were analysed similarly to Dataset 1 but using SPM (www.fil.ion.ucl.ac.uk/spm). fMRI volumes were spatially realigned for motion correction, normalized to standard MNI space, re sampled to 3 × 3 × 3 mm voxel size, and then spatially smoothed (FWHM = 5 mm) and temporally high-pass filtered (1/128 Hz cutoff). For each participant, first-level statistical parametric maps were obtained using a GLM with regressors modeling the occurrence of each of the five conditions (pain trials matched with touch, touch trials matched with pain, unmatched pain trials, unmatched touch trials, rating period), their corresponding temporal derivatives, and additional regressors corresponding to the head motion parameters. A contrast analysis was performed in each participant to identify voxels showing significant differences in the fMRI responses to painful and tactile stimuli. A second-level analysis (one-sample two-tailed *t*-test) was then performed to obtain group-level statistical parametric maps. Voxels showing significant differences between pain and touch were identified using a cluster-level (cluster-defining threshold of *P* < 0.001) family-wise-error (FWE) corrected threshold of *P* < 0.05.

As Datasets 1 and 2 were collected and analysed for different purposes, the processing pipelines described above were not identical. Therefore, to test whether the GLM results depended on the actual processing pipeline, we also performed a second univariate GLM analysis of both datasets processed using the same pipeline and software (see Supplementary Information for detailed methods and results).

### Univariate ROI-wise Analysis

Very stringent statistical thresholds were unavoidable in the above mass-univariate GLM analysis due to the large number of voxels in the pre-selected “pain matrix” mask. To reduce the multiple comparison problem so as to increase the statistical power of detecting differences in fMRI responses elicited by different stimuli, we also tested whether differences in the average BOLD signals in the “pain matrix” related brain areas could be detected using ROI-based univariate analysis. An important advantage of such ROI-based analyses is that, in contrast with the GLM, it does not make any assumption about the shape of the hemodynamic response function (HRF). Ten ROIs were selected for this analysis (ROI locations are shown in Fig. 1): the left and right S1, the left and right S2, the left and right insula, the left and right anterior/mid cingulate cortex, and the left and right thalamus. The “S1 foot” ROI used during the selection of the “pain matrix” mask was split into two halves according to the *x*-axis coordinates: the voxels with *x* ≤ 0 as the left S1 ROI and the voxels with *x *> 0 as the right S1 ROI. The other eight ROIs were defined by the overlap between the “pain matrix” mask and the corresponding brain areas defined in the AAL template (Tzourio-Mazoyer et al. 2002): the left and right Rolandic operculum (corresponding to S2), the left and right insula, the left and right anterior/mid cingulate, and the left and right thalamus.

#### Dataset 1

After the 10 ROIs were defined, the preprocessed data used in the subsequent MVPA were used to calculate the average BOLD signal across all voxels for each ROI and for each of the 56 samples (see details for the preprocessing steps for MVPA on Dataset 1 in the following subsection “multivariate pattern analysis: nociceptive vs. non-nociceptive stimuli”) to provide a direct comparison with the subsequent MVPA results. For each of the 10 ROIs and each of the three comparisons (“pain vs. touch,” “pain vs. audition” and “pain vs. vision”), a paired *t-*test was performed to test whether the average BOLD signals were significantly different between different eliciting stimuli in the sub-group of subjects selected for matching the stimulus saliency for the given comparison. To address the problem of multiple comparisons, a non-parametric permutation test (*n* = 5 000) was performed to determine the statistical significance. To further examine how stimulus-evoked BOLD responses unfolded over time in each ROI, we extracted the time courses of the BOLD responses averaged across trials, separately for the pain and touch conditions, using a time window of 18 s (i.e., from −1 TR to +5 TR with respect to stimulus onsets).

#### Dataset 2

The same ROI-wise analyses were performed on the preprocessed data used in the subsequent MVPA of Dataset 2. Details of the preprocessing for the MVPA “pain vs. touch” on Dataset 2 are in the following section (“Multivariate pattern analysis: nociceptive vs. non-nociceptive stimuli”). To further examine how stimulus-evoked BOLD responses unfolded over time in each ROI, we extracted the time courses of the BOLD responses averaged across trials, separately for the pain and touch conditions, using a time window of 12.8 s (i.e., from −1 TR to +15 TR with respect to stimulus onset).

### Multivariate Pattern Analysis: Nociceptive vs. Non-nociceptive Stimuli

MVPA is a machine learning technique that uses a pattern classifier (Mur et al. 2009; Pereira et al. 2009) to identify the representational content of the neural responses elicited by different stimuli. In contrast with the univariate analyses which detect regional averaged activations and consider a single-voxel or a single ROI at a time, MVPA analyses the spatial pattern of fMRI signals across all voxels within a pre-defined area. That is, MVPA detects condition-specific patterns of activity across many voxels at once. Whereas GLM directly compares differences in signal amplitude on a voxel-by-voxel basis, MVPA projects samples composed by multiple voxels from each condition of interest into a high dimensional space, and searches for the boundary between the samples from two or more conditions (Mur et al. 2009). MVPA is usually more sensitive than conventional univariate analysis (i.e., GLM) in disclosing differences in brain activities between experimental conditions not only because it offers a powerful solution to the problem of multiple comparisons, but also because it performs a joint analysis of patterns of activity distributed across multiple voxels. In the present study, MVPA was performed using the PyMVPA software package (Hanke et al. 2009), in combination with LibSVM’s implementation of the linear support vector machine (SVM; www.csie.ntu.edu.tw/~cjlin/libsvm).

#### Dataset 1

Before MVPA, fMRI data were preprocessed using FSL (www.fmrib.ox.ac.uk/fsl) and Matlab (Mathworks; www.mathworks.co.uk). After the same motion correction performed for the mass-univariate GLM analysis, fMRI data were neither spatially smoothed nor temporally filtered, but further corrected for head motion by regressing out the six motion parameters estimated during the spatial alignment from the time series of each voxel. The head motion was summarized as an average displacement during the stimulation of each modality calculated from the six motion parameters (Jones et al. 2008; Birn et al. 2014), and then compared between the two sensory modalities in each classification using paired *t*-tests. The results confirmed that the head motion parameters were not significantly different across sensory modalities (Table 1). For each run, the time series of each voxel were linearly detrended and converted to *Z*-scores by subtracting the mean and dividing by the standard deviation across all time points of the time series. Given that the second volume after each stimulus onset (i.e., the volume acquired 4–6 s after stimulus onset) corresponded approximately to the peak of the BOLD signal elicited by each stimulus and was thus the most likely to contain stimulus-related information (Liang et al. 2013) and the peak latencies were very similar across modalities (Mouraux et al. 2011), these volumes were averaged across all trials, separately for each sensory modality, run and subject. This resulted in four volumes, one for each modality, in each run and subject. The volumes were then normalized to standard MNI space and re sampled to 2 × 2 × 2 mm voxel size. Finally, the volumes belonging to the same modality were further averaged across runs for each subject, resulting in four average volumes (one for each sensory modality) in each subject.

To test whether successful classifications were contributed by possible differences in the mean signal amplitude of the ROI between different stimuli (including differences due to their peak latencies), the following classification analyses were also performed after signal normalization, obtained by subtracting from the signal of each voxel the mean signal across all voxels of the “pain matrix” ROI and then dividing the result by the standard deviation of the signal from all voxels of the ROI (Hu and Iannetti 2016). Consequently, for each volume, all voxels within the ROI had a mean of 0 and a standard deviation of 1.

We performed three between-subject classifications to distinguish fMRI responses elicited by painful stimuli from those elicited by non-painful stimuli (i.e., “pain vs. touch,” “pain vs. audition” and “pain vs. vision”). The same sub-group of subjects selected for each univariate comparison was used in each classification (i.e., *N* = 10 for “pain vs. touch,” *N* = 10 for “pain vs. audition,” and *N* = 8 for “pain vs. vision”). A “leave-one-subject-out” cross-validation approach was employed to train and test the classifier algorithm. For an *N*-fold cross-validation, in each cross-validation step, the classifier was trained on *N* − 1 subjects and tested on the *N*th subject. This procedure was repeated *N* times, using each time a different subject as test dataset. In each cross-validation step, the classifier performance was calculated as classification accuracy, i.e., the number of correct guesses divided by the number of test samples. The overall performance in each classification was derived by averaging the classification accuracies obtained from all cross-validation steps.

We also generated sensitivity maps for each two-way classification. In these maps, the value of any given voxel represents its linear SVM weight. A high weight implies that the voxel gave a strong contribution to the classifier’s accuracy in predicting whether the eliciting stimulus was painful or not. The sign of the SVM weight indicates the preference for pain or the other non-painful modality being compared in each two-way classification. That is, when considering the classification “pain vs. *X*,” where *X* is one of the three non-painful modalities, a positive weight implies that the voxel exhibits a higher BOLD signal during pain compared to *X*, whereas a negative weight implies that the voxel exhibits a higher BOLD signal during *X* compared with pain.

To test whether the classification accuracy was higher than chance level (i.e., 0.5 in our case), we built the null distributions of the classification accuracies under the scenario in which each average volume was labeled randomly and thus did not contain any information about the modality of the stimulus eliciting the recorded fMRI response when training the classifier. Such permutation testing (*n* = 5 000) was performed for each classification and for the training dataset only. The non-parametric *P*-value was then derived for each classification by comparing the actual classification accuracy obtained from the correctly labeled data and the null distribution, that is, the proportion of the null distribution that is equal to or higher than the actual classification accuracy.

The same MVPA analyses were also performed using the signals from the two “non-pain matrix” masks to test whether the voxels outside the selected “pain matrix” also contain pain-specific information that allows successful discrimination between pain and the other three sensory modalities.

#### Dataset 2

As only painful and tactile stimuli were delivered in Dataset 2, we performed the classification “pain vs. touch.” fMRI data were preprocessed as for the GLM analysis, except that the data were not spatially smoothed or temporally filtered. Data were corrected for head motion by regressing out the six motion parameters estimated during the spatial alignment from the time series of each voxel and linearly detrended for each session. For this MVPA, we used the subset of trials that were selected in each participant to match the perceived intensity of painful and tactile stimuli as described in the GLM analysis of Dataset 2. Given that the eighth volume after each stimulus onset was acquired approximately 6 s after stimulus onset, i.e., at the expected peak of the stimulus-evoked BOLD response, these volumes were averaged across all selected trials, separately for each sensory modality and participant. This resulted in two average volumes for each participant, one for each modality.

The same “leave-one-subject-out” cross-validation approach was used for the classification performed in Dataset 2. The same permutation test (*n* = 5 000) was used for determining the significance of the classification accuracy. Sensitivity maps were also generated.

#### Cross-datasets Classification

To test the generalizability of the patterns underlying the two within-dataset classifications, we performed two across-datasets classifications: (1) testing on Dataset 1 using the model trained on Dataset 2, and (2) testing on Dataset 2 using the model trained on Dataset 1. Separate classification accuracies and sensitivity maps were obtained for these two classifications. This across-datasets classification is more challenging than within-dataset classification as the two datasets were collected using different acquisition parameters from two different scanners, and therefore the properties of their fMRI signal (e.g., their amplitude at baseline) and noise were likely to be different. For this reason, only normalized signals were used for the across-datasets classification. Furthermore, differences in experimental design (e.g., event-related design for Dataset 1 and blocked design for Dataset 2; see Methods and Supplementary Fig. S2) could have determined differences in the response to the same stimuli. This represents another challenge for the across-datasets classification.

#### Classifications Within Sub-regions of “Pain Matrix”

All classifications described above exploited information from the entire “pain matrix,” i.e., both the spatial patterns of the fMRI signals “within” each “pain matrix” sub-region and the spatial patterns across different “pain matrix” sub-regions. To test whether within-region classifications were also possible, we performed the same within-dataset and across-datasets classification analyses using only the signals within each of the 10 sub-regions defined in the “univariate ROI-wise analysis.”

#### Classifications using GLM Beta Maps

All classification analyses described above exploited the BOLD signals sampled at the “peak” of the BOLD response (i.e., they used the fMRI volumes capturing the response peaks) averaged across trials. One advantage of this approach is that it avoids the assumption about the shape of the HRF. However, BOLD responses may have different peak latency for different stimulus types and in different brain regions. To rule out the possibility that MVPA results were affected by possible differences in the latency of the BOLD responses to pain and touch, we repeated the within-dataset and across-datasets classification analyses using the beta maps obtained in the mass-univariate GLM analysis.

### Multivariate Pattern Analysis: High- vs. Low-Intensity/Saliency Stimuli

#### Dataset 1

To test whether the BOLD signals sampled from the same set of voxels (i.e., the “pain matrix” mask) contain information about the stimulus saliency regardless of stimulus modality, a similar MVPA analysis was performed using the “pain matrix” mask to distinguish the BOLD signals elicited by high-saliency stimuli and those elicited by low-saliency stimuli using the same procedure described above except the following differences: the 56 preprocessed samples (4 modalities × 14 subjects) were sorted by their saliency regardless of sensory modality, and re-assigned to one of the two classes (“high-saliency” and “low-saliency”) by median split. This resulted in 10 pain samples, six touch samples, seven audition samples and five vision samples in the “high-saliency” class and four pain samples, eight touch samples, seven audition samples and nine vision samples in the “low-saliency” class. To remove any difference in the number of samples of each sensory modality between the two classes, the number of samples was balanced across the two classes for any given sensory modality by removing six pain samples with relatively low-saliency from the “high-saliency” class and removing two touch samples and four vision samples with relatively high-saliency from the “low-saliency” class, resulting in 44 samples in total (22 samples in each class including 4 pain samples, 6 touch samples, 7 audition samples, and 5 vision samples) for the MVPA analysis. The same MVPA procedure described in the pain-related classifications was performed to obtain the classification accuracy and the corresponding sensitivity map.

#### Dataset 2

All trials from both sensory modalities and all participants were pooled and sorted from low to high according to the perceived stimulus intensity, regardless of stimulus modality, and then median split into two classes (high and low-intensity). The trials from one participant were discarded as they were all assigned to the “high” group. All remaining trials were averaged for each sensory modality, participant and group, resulting in 100 samples in each group (one sample for each of the two sensory modalities and each of the 50 participants). Thus, the number of samples were matched between groups (high, low) for sensory modality and participant. The same procedure described for the classification of Dataset 1 was performed to obtain the classification accuracy and the corresponding sensitivity map.

#### Across-Datasets Classification

We performed the same across-datasets classification described previously, but for distinguishing high versus low-intensity/saliency stimuli. Classification accuracies and sensitivity maps were obtained separately for the classifier trained using Dataset 2 and tested using Dataset 1, and vice versa.

## Results

### Univariate Analyses Require Large Sample Sizes to Detect Pain-Selective Neural Activity Across Individuals

#### Mass-Univariate GLM Analysis of Dataset 1

In a preliminary control analysis, we used standard mass-univariate GLM analysis to directly compare the fMRI responses elicited by transient painful stimuli with those elicited by equally-salient but non-nociceptive and non-painful tactile, auditory and visual stimuli with carefully matched stimulus saliency. This comparison was performed for the voxels within the “pain matrix” ROI (Fig. 1). We found that GLM results depended considerably on the type of data processing pipeline: there were no difference between pain and non-pain conditions when using the FSL software, even with the liberal FDR correction, or when using SPM8 with voxel-based FWE correction. However, when using SPM8 with cluster-based FWE correction, several clusters in the bilateral thalamus and insula revealed higher activation for pain than for touch, and one cluster in the left posterior insula revealed higher activation for pain than for vision (see Supplementary Fig. S7). This result indicates that the identified differences between pain and non-pain conditions do not seem very reliable with a sample size of *n* ≤ 14.

#### Mass-Univariate GLM Analysis of Dataset 2

**Figure 2.**
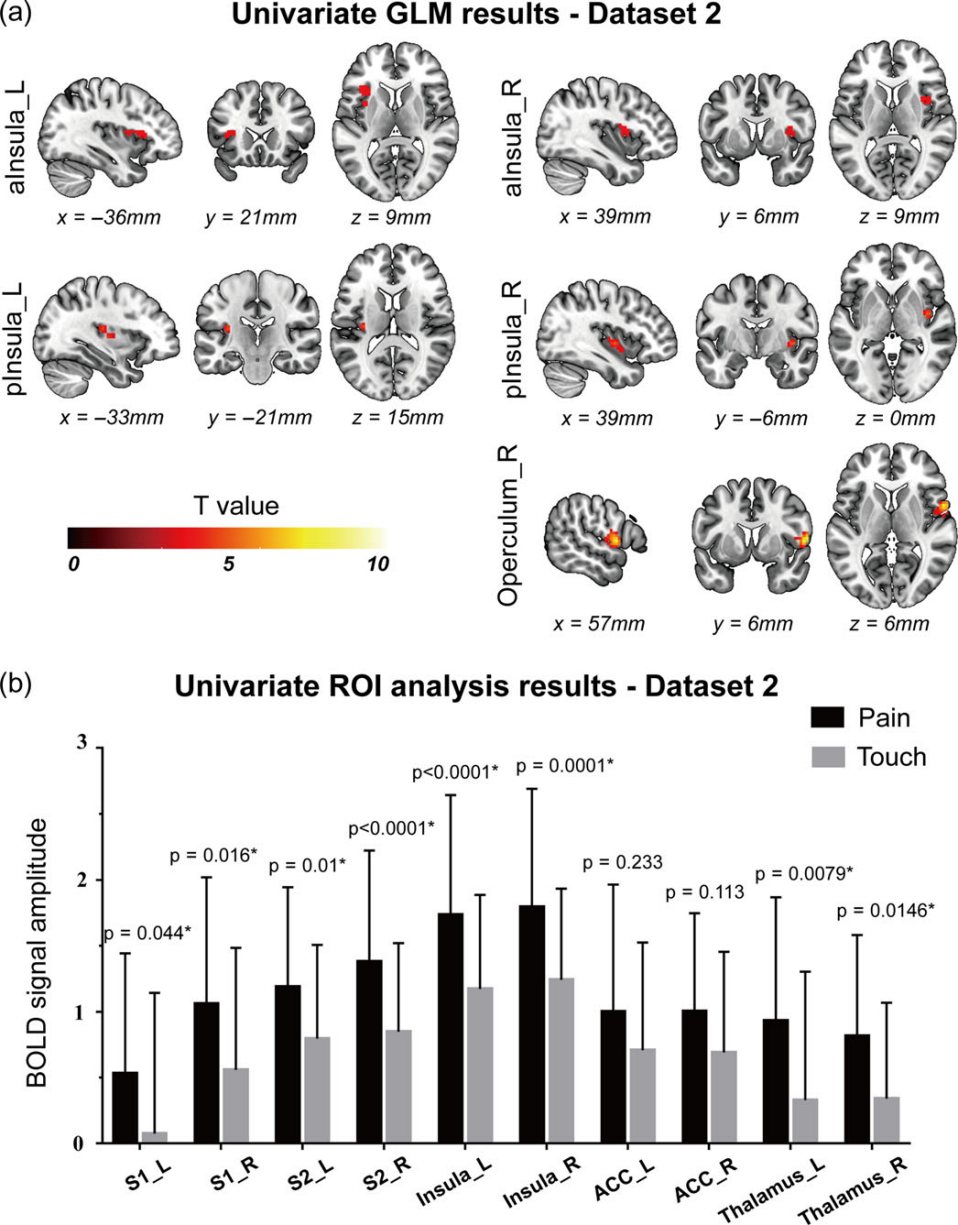
Results of univariate GLM analysis (**a**) and ROI-wise analysis (**b**) obtained from Dataset 2. Panel **a**: five clusters in the bilateral insula (including both anterior and posterior part) and the right operculum (S2) were detected by GLM analysis to have stronger responses to painful stimuli than to tactile stimuli, and no voxel was detected to have higher responses to tactile stimuli than to painful stimuli. Panel **b**: BOLD signals and corresponding *P*-values of “pain vs. touch” comparison (paired *t*-test) for all explored brain regions. All regions except the bilateral ACC showed significantly higher responses to painful stimuli than to tactile stimuli. The BOLD signal amplitudes are shown as the average and standard deviation across participants. *P-*values < 0.05 are indicated by asterisks. L: left; R: right; aInsula: anterior insula; pInsula: posterior insula.

The results of Dataset 2, which entailed a much larger sample size (*n* = 51 in Dataset 2 vs. *n* = 14 in Dataset 1), looked more stable with respect to the type of processing pipeline used. After a smoothing of 5 mm Gaussian kernel and cluster-based FWE correction, some voxels in the bilateral insula and the right S2 showed significantly stronger responses to pain than to touch (Fig. 2). When increasing the smoothing kernel to 8 mm, stronger responses to pain than to touch were still detected in the right S2 for both cluster-based and voxel-based FWE correction, and in two small clusters in the left anterior insula and S2, but only for voxel-based FWE correction (see Supplementary Fig. S7).

#### ROI-wise Univariate Analysis of Dataset 1

**Figure 3.**
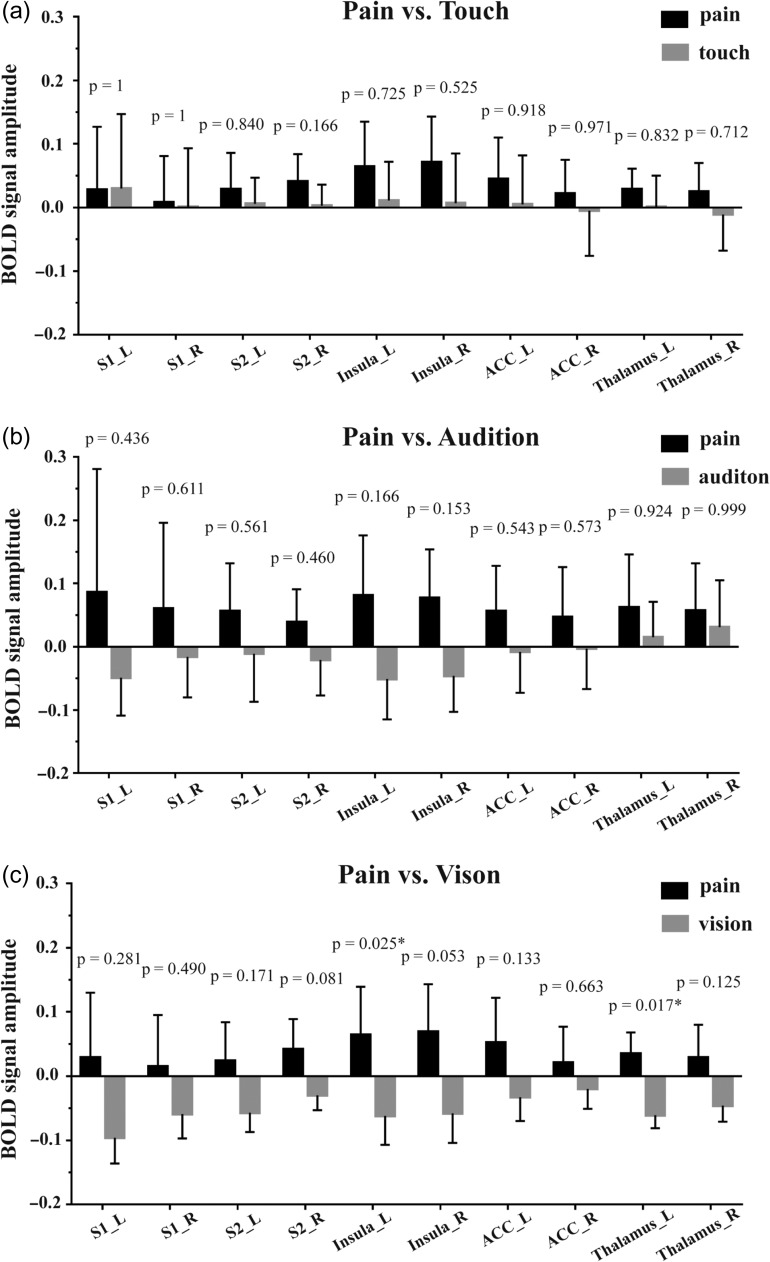
BOLD signal amplitude in all explored brain regions, along with their corresponding *P-*values for the three comparisons between the modalities of the eliciting stimuli: pain vs. touch (**a**), pain vs. audition (**b**) and pain vs. vision (**c**). The BOLD signal amplitude are shown as the average and the standard deviation across participants. *P-*values < 0.05 are indicated by asterisks. L: left; R: right.

For each of the 10 pre-defined ROIs (Fig. 1), we compared the response amplitude (after having matched stimulus saliency) in the following two-way comparisons: “pain vs. touch,” “pain vs. audition” and “pain vs. vision.” No ROI displayed significantly different responses in the “pain vs. touch” and “pain vs. audition” comparisons (Fig. 3a&b). Two ROIs showed significantly different BOLD response magnitudes in the “pain vs. vision” comparison: nociceptive stimuli elicited stronger responses than visual stimuli in the left insula (*P* = 0.025) and the left thalamus (*P* = 0.017) (Fig. 3c). The time courses of BOLD signals in each ROI and condition are shown in Supplementary Fig. S8a.

#### ROI-wise Univariate Analysis of Dataset 2

The same ROI-wise analyses were repeated to compare the response amplitude between pain and touch, after perceived stimulus intensity was carefully matched across the two modalities. The results show that eight out of ten ROIs showed significantly stronger responses to pain than to touch (*P* < 0.05; Fig. 2b). Only the bilateral ACC did not show significant difference in response amplitude between pain and touch (*P* > 0.1; Fig. 2b). This result is in contrast with the results obtained from Dataset 1, suggesting that a large sample size is needed for univariate analysis to detect robust differences in fMRI responses between pain and touch. The time courses of BOLD signals in each ROI and condition are shown in Supplementary Fig. S8b.

### MVPA can Detect Pain-related Patterns of Brain Activity Across Individuals

#### Dataset 1

**Figure 4.**
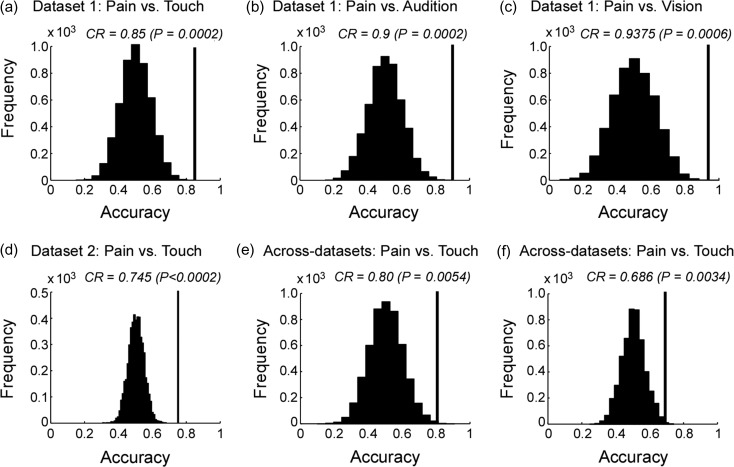
Within-dataset (**a–d**) and across-datasets (**e**, **f**) classification accuracies of “pain vs. non-pain” classifications obtained from normalized data, along with the corresponding null distributions. Panels **a–c**: results obtained from Dataset 1 for the three classifications, respectively. Panel **d**: result obtained from Dataset 2 for the “pain vs. touch” classification. Panel **e**: result obtained using Dataset 2 as training dataset and Dataset 1 as test dataset. Panel **f**: result obtained using Dataset 1 as training dataset and Dataset 2 as test dataset. Classification accuracies (correct rate, CR) are indicated by black vertical lines and corresponding null distributions (obtained from 5 000 permutations) are indicated by black bell shapes centered around chance level accuracy of 0.5. *P*-values were calculated as the proportion of how many (out of 5 000) permutations generated accuracy greater than or equal to the actual classification accuracy. If none out of 5 000 permutations reached the actual accuracy, the *P*-value is labeled as *P* < 0.0002 (i.e., <1/5 000).

Three two-way classifications to compare fMRI responses elicited by saliency-matched painful and non-painful stimuli (“pain vs. touch,” “pain vs. audition” and “pain vs. vision”) were performed. All classifications showed that the information contained in the spatial distribution of the fMRI signals sampled from the “pain matrix” ROI allowed distinguishing painful nociceptive stimuli from equally-salient, but non-painful tactile, auditory and visual stimuli. The obtained accuracies of the three classifications are shown in Fig. 4a-c (for normalized data) and Supplementary Fig. S9a-c (for non-normalized data), along with the corresponding null distributions. These findings indicate that the classification accuracies were not solely contributed by differences in the mean signal amplitude of the ROI between different stimuli and, instead, that the spatial distributions of the fMRI responses were sufficiently dissimilar to correctly distinguish brain activity elicited by painful stimuli from that elicited by equally-salient, non-painful, stimuli. Because stimuli were matched in terms of subjective saliency, the classifications were unlikely to have been driven by differences in stimulus saliency (Iannetti et al. 2008).

The sensitivity maps showing the spatial distribution of the voxels contributing to the classifications are displayed in Supplementary Fig. S10. These maps reveal that the voxels contributing to each classification were clustered rather than scattered. This indicates that the mesoscopic pattern of activity within and across the brain regions composing the so-called “pain matrix” allowed the classifier to predict correctly the modality of the eliciting stimulus.

**Figure 5.**
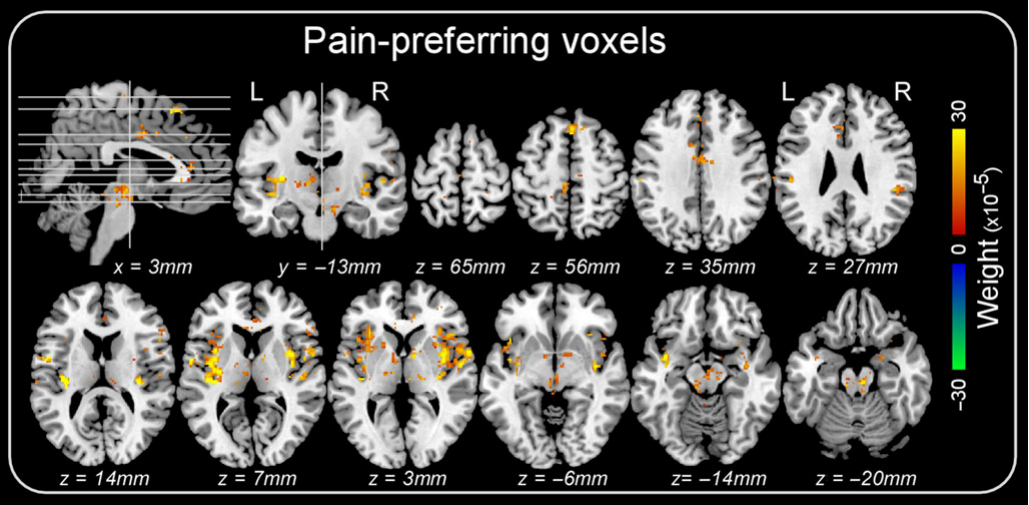
Voxels consistently showing higher BOLD signal during pain across all three classifications (“pain vs. touch,” “pain vs. audition” and “pain vs. vision”) in Dataset 1. Colors code the average weight across the three classifications.

We also identified voxels consistently showing a response preference for pain (i.e., voxels with positive weights) in all three classification tasks. These “pain-preferring” voxels were located in the brainstem, thalamus, insula, anterior and mid cingulate cortex, and supplementary motor area (Fig. 5).

To test whether voxels outside the “pain matrix” ROI also contain information that allows successful discrimination between saliency-matched painful and non-painful stimuli, we repeated the same MVPA classifications (“pain vs. touch,” “pain vs. audition” and “pain vs. vision”) on a “non-pain-matrix” mask (Supplementary Fig. S3a) containing all voxels not included in the “pain matrix” ROI. These results showed that the classification accuracies of “pain vs. audition” (correct rate = 0.90, *P* = 0.0002) and “pain vs. vision” (correct rate = 1, *P* < 0.0002) were significantly higher than chancel level (Supplementary Fig. S3c&d). This observation was expected, as both auditory and visual cortices were included in this mask (although the correct classification could have been also contributed by neural activity outside auditory and visual cortices). In contrast, the classification accuracy of “pain vs. touch” was around chance level (correct rate = 0.55, *P* = 0.408; Supplementary Fig. S3a). Using the second “non-pain-matrix” mask having the same number of voxels as the “pain matrix” mask (Supplementary Fig. S4a), we observed similar results (Supplementary Fig. S4b–d): correct rate = 0.45 (*P* = 0.79) for “pain vs. touch,” correct rate = 0.75 (*P* = 0.012) for “pain vs. audition,” correct rate = 0.9375 (*P* = 0.0006) for “pain vs. vision”.

#### Dataset 2

The “pain vs. touch” classification performed using Dataset 1 was repeated using Dataset 2 (which only included painful and tactile stimuli), and similar results were obtained (Fig. 4d and Supplementary Fig. S9d). The fMRI signal within the “pain matrix” ROI again correctly predicted painful versus tactile stimuli (*P* < 0.0002 for both non-normalized and normalized signals; 5 000 permutations; see Fig. 4d and Supplementary Fig. S9d). The corresponding sensitivity map obtained from the normalized data is shown in Supplementary Fig. S11a.

However, and in contrast with the result obtained in Dataset 1, the classification accuracies obtained from Dataset 2 using the two “non-pain matrix” masks were significantly higher than chance level (Supplementary Figs S3e and S4e). The sensitivity map corresponding to the “non-pain matrix” Mask 1 showed that several brain areas outside the “pain matrix” contributed more than others to the successful classification (Supplementary Fig. S11b): for example, a cluster located in bilateral paracentral lobule/supplementary motor areas (i.e., the foot area of the primary sensorimotor cortex) had higher signal during painful stimulation versus tactile stimulation; and a cluster in the left lateral postcentral gyrus (i.e., contralateral to the stimulated foot) had higher signal during tactile stimulation versus painful stimulation.

#### Classification Across-Datasets

Despite more challenging (see details in Methods), the across-datasets classification still showed a good accuracy (Fig. 4e–f): correct rate = 0.80 (*P* = 0.0054) when the classifier was trained using Dataset 2 and tested on Dataset 1; and correct rate = 0.69 (*P* = 0.0034) when the classifier was trained using Dataset 1 and tested on Dataset 2. These results indicate that the patterns identified in each dataset were generalizable to another independent dataset.

**Figure 6.**
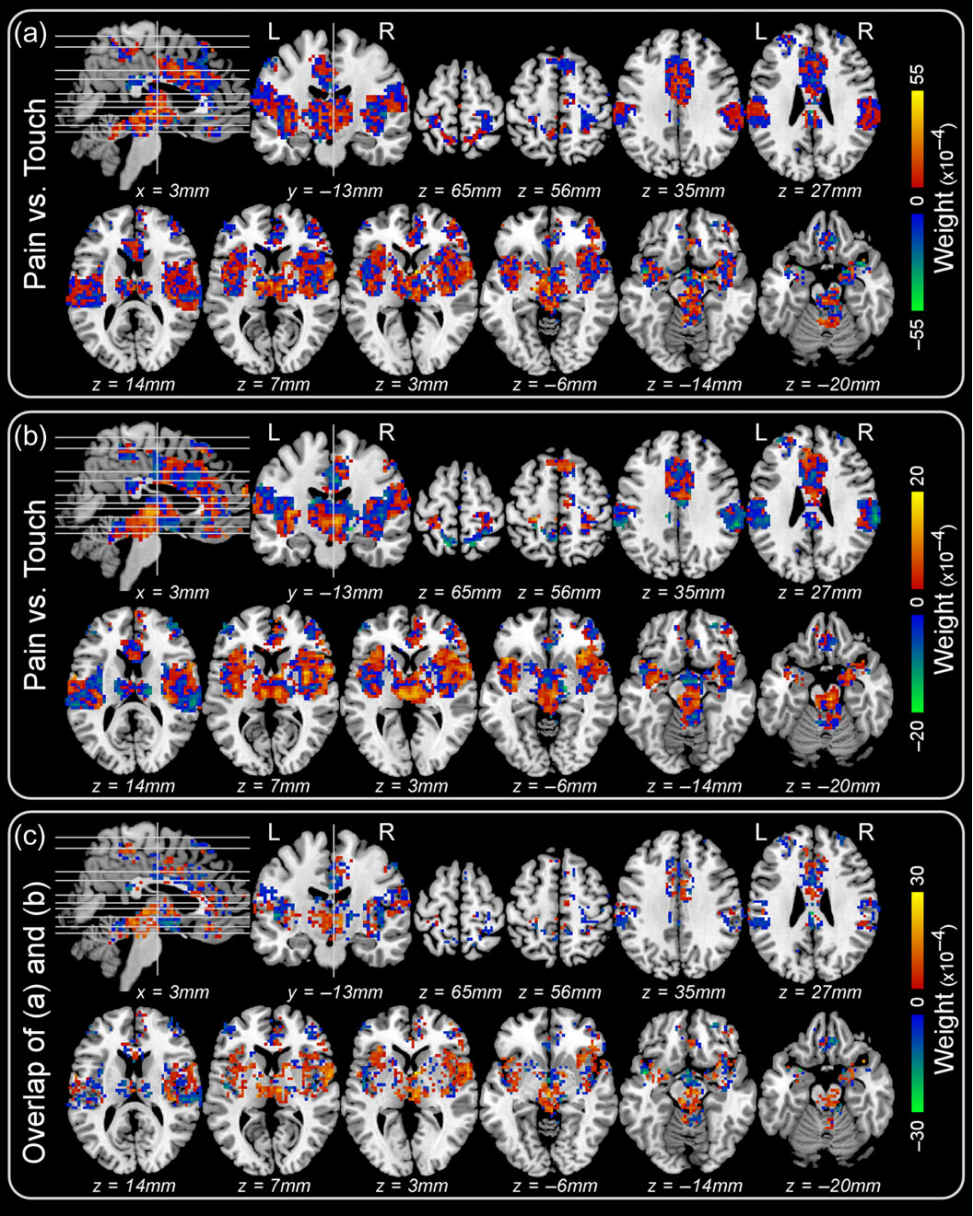
Sensitivity maps obtained from the “pain vs. touch” classification across-datasets. Panel **a**: sensitivity map obtained when the classifier was trained using Dataset 2 and tested on Dataset 1. Panel **b**: sensitivity map obtained when the classifier was trained using Dataset 1 and tested on Dataset 2. Panel **c**: overlap (i.e., the voxels of which the weights have consistent sign) between the two sensitivity maps (**a**) and (**b**).

The sensitivity maps were also generated from the two across-datasets classifications (Fig. 6a&b), and the voxels with consistent weight sign across the two sensitivity maps are shown in Fig. 6c.

We also tested the classification across-datasets using the two “non-pain-matrix” masks. The resulting four classifications were not successful (Supplementary Figs S3f–g and S4f–g).

#### Classifications within Individual “Pain Matrix” Sub-regions

The within-dataset and across-datasets classifications obtained using the fMRI signal from individual “pain matrix” sub-regions are showed in Supplementary Figs S12 and S13, respectively. Although within-dataset classifications within a few individual sub-regions (e.g., the thalamus, the S2 and the insula) were successful using one dataset, they were not successful using the other dataset (Supplementary Fig. S12). None of these individual sub-regions showed successful classifications in the across-datasets classification (Supplementary Fig. S13).

#### Classifications using GLM Beta Maps

Pain and touch conditions could be distinguished within Dataset 1, within Dataset 2, and across datasets, when using beta maps of the entire “pain matrix” derived from univariate GLM analyses (see Supplementary Fig. S14). The within-region classification results also remained the same when beta maps were used (Supplementary Figs S15 and S16). These results, together with the successful classifications using BOLD signals, indicate that the distinguishable “pain vs. touch” activity patterns within the “pain matrix” were not simply due to this latency difference.

### MVPA can Detect Intensity/Saliency-related Patterns of Brain Activity Across Individuals

#### Dataset 1

**Figure 7.**
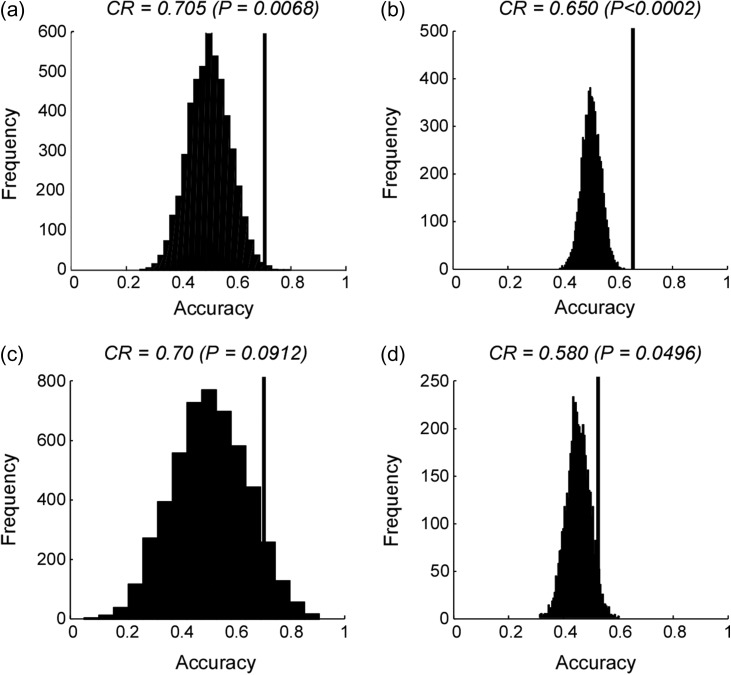
Within-datasets (**a**, **b**) and across-datasets (**c**, **d**) classification accuracies of “high vs. low-intensity/saliency” classification obtained from normalized data, along with the corresponding null distributions. Panels **a** and **b**: results obtained from Dataset 1 and Dataset 2, respectively. Panel **c**: result obtained using Dataset 2 as training dataset and Dataset 1 as test dataset. Panel **d**: result obtained using Dataset 1 as training dataset and Dataset 2 as test dataset. Classification accuracies (correct rate, CR) are indicated by black vertical lines and corresponding null distributions (obtained from 5 000 permutations) are indicated by black bell shapes centered around chance level accuracy of 0.5. *P*-values were calculated as the proportion of how many (out of 5 000) permutations generated accuracy greater than or equal to the actual classification accuracy. If none out of 5 000 permutations reached the actual accuracy, the *P*-value is labeled as *P* < 0.0002 (i.e., <1/5 000).

When classifying between “high-saliency” stimuli and “low-saliency” stimuli regardless of their sensory modality, the resulting classification accuracy was significantly higher than chance level (see Fig. 7 for normalized data and Supplementary Fig. S17 for non-normalized data).

#### Dataset 2

We obtained similar results when the same high versus low-intensity classification was performed using Dataset 2: classification accuracies obtained using both normalized and non-normalized signals were significantly higher than chance level (Fig. 7 and Supplementary Fig. S17).

#### Classification Across-Datasets

The spatial patterns of fMRI signal allowing the distinction between high- and low-intensity/saliency obtained separately from the two datasets were less generalizable than those allowing the distinction between pain and non-painful sensations: when the classifier was trained using Dataset 2 and tested on Dataset 1, the classification accuracy was 0.7, with a significance level of only *P* = 0.0912; when the classifier was trained using Dataset 1 and tested on Dataset 2, the classification accuracy was 0.58, with a significance level of *P* = 0.0496 (Fig. 7).

Sensitivity maps of the within-dataset and across-datasets classifications are shown in Supplementary Fig. S18.

## Discussion

Is there pain-selective information within the fMRI responses elicited by painful stimuli after controlling for stimulus intensity/saliency? Here, we addressed this question by performing between-subject MVPA of the fMRI responses elicited by a range of “equally-intense” and “equally-salient” nociceptive, tactile, auditory, and visual stimuli in two independent datasets collected from different MRI scanners. We were thus able to perform both within-dataset classifications and across-datasets classifications.

In both datasets, we observed that spatial patterns of fMRI signal allowed distinguishing the responses elicited by a transient painful nociceptive stimulus from those elicited by “equally-intense” and “equally-salient” non-painful stimuli. Such spatial patterns were consistent across-datasets collected in different MRI scanners. We also found that spatial patterns of fMRI signals were able to distinguish the responses to high- vs. low-intensity/saliency stimuli, regardless of their sensory modality.

### Brain Responses Distinguishing the Modality of Intensity/Saliency-Matched Stimuli

We first performed a GLM analysis to test whether it was possible to identify clusters of voxels showing significantly different levels of activation during nociceptive stimulation eliciting pain compared with other stimulations not eliciting pain. To ascertain the robustness of the results, fMRI data were processed using different pipelines and software (see Supplementary Methods for details). In Dataset 1 (*n* = 14), results were not very robust, as differences in activation between pain and non-pain conditions were detected using one processing pipeline, whereas no difference was detected using the other two processing pipelines (Supplementary Fig. S7). Furthermore, in the ROI-based raw-signal analyses, none of the 10 “pain matrix” ROIs showed clear differences in the overall average BOLD signals for all three comparisons between painful and non-painful stimuli (Fig. 3). In Dataset 2 (*n* = 51), where the statistical power is increased because of the much larger sample size, results were more robust, and differences in activation between pain and touch were detected using all three processing pipelines: the right S2 always showed stronger responses to painful stimuli than to tactile stimuli (Figs 2a and Supplementary Fig. S7). All 10 “pain matrix” ROIs, with the notable exception of the bilateral ACC, had stronger responses to painful stimuli than to tactile stimuli (Fig. 2b). These results suggest that univariate GLM results depends considerably on the processing pipeline, especially when the sample size is small, such as in Dataset 1.

Note that differences in the spatial patterns of fMRI responses detected by MVPA across individuals necessarily implies the existence of some univariate differences in signal amplitude. Our results indicate that such univariate differences are subtle, and require a large sample to be detected by standard univariate analysis while controlling for false positive rate at a reasonable level (Figs 2 and 3 and Supplementary Fig. S7). This result provides important additional information to our previous report that salient stimuli of four sensory modalities activated the same set of voxels, but without explicitly testing whether the intensity of activation within these voxels was different between pain and other modalities after matching stimulus intensity/saliency across participants (Mouraux et al. 2011). Several previous studies have suggested pain specificity of the dorsal posterior insula and the operculum, using a variety of neuroimaging techniques such as PET, fMRI, ERPs, intracerebral recordings (Peyron et al. 2002), arterial spin labeling (Segerdahl et al. 2015), as well as electrical stimulation of the operculoinsular cortex (Ostrowsky et al. 2002; Afif et al. 2010; Isnard et al. 2011). However, most of these previous studies suffer from the same problem of lacking an adequate control stimulus matched in term of saliency or intensity (Mouraux and Iannetti 2018).

By performing MVPA on both Datasets 1 and 2, we observed that the spatial patterns of the fMRI responses elicited by painful stimuli within the so-called “pain matrix” were distinguishable from those elicited by non-painful, but equally-intense and equally-salient, tactile, auditory and visual stimuli (Fig. 4a–d and Supplementary Fig. S9). This result was further corroborated by the observation that these patterns are stable across different datasets collected using different parameters and scanners (Fig. 4e–f). Notably, this classification was successful only when using the signals from the “pain matrix” but not when using the signals from individual sub-regions of the “pain matrix” (Supplementary Figs S12, S13, S15 and S16). These findings, together with our previous results (Mouraux et al. 2011), indicates that even if (1) the voxels activated by transient painful and non-painful sensory stimuli are virtually the same (Mouraux et al. 2011), and (2) the univariate differences in signal intensity are not strong enough to be reliably detected by conventional GLM analysis, the spatial patterns of fMRI signal across voxels widely distributed within the “pain matrix” (but not within a local sub-region), are consistently different between painful stimuli and non-painful stimuli, across participants. Our result agrees with the view that pain, as any other subjective experience, is likely to emerge from a specific pattern of neural activity, which has been postulated as a “neuromatrix” (Melzack 1989, 2001; Iannetti and Mouraux 2010) and, more recently, as a “pain connectome” (Kucyi and Davis 2015). In this view, the experience of pain would not be a result of the mere activation of certain brain areas or neurons. Instead, pain would be an “emergent property” arising from the synchronized or coordinated activity of multiple brain areas which, if considered in isolation, are not specific for pain.

All results reported here were obtained by performing a “between-subject” MVPA. The rationale for this choice was that, within the context of Dataset 1, only between-subject MVPA permitted for a good matching of subjective saliency in each of the three pairs of comparisons, thus allowing proper inferences about response selectivity (Hu and Iannetti 2016). Unlike “within-subject” MVPA, which examines whether different stimulus categories have reliably distinct cortical representations at the level of single subjects, between-subject MVPA requires the spatially distinct representations to be consistent across subjects. Therefore, the observed successful classifications implies that specific patterns, although likely to be shaped by individual experiences, are at least partly innate and preserved across individuals.

That the identified patterns of neural activities can be generalized to different subjects implies that they are rather coarse in their spatial scale. Indeed, fine-grained differences are unlikely to be detectable using between-subject MVPA, given (1) the functional and anatomical differences between individuals, and (2) that the anatomical coregistration of brains from different individuals does not allow spatial matching at voxel level. Therefore, classification using between-subject MVPA can only rely on spatial patterns occurring at a spatial scale between single-voxel patterns and regional brain activity. For this reason, the voxels that contributed most to the classification of painful versus non-painful stimuli formed clear clusters, located in the insula, the anterior and mid cingulate cortex, the supplementary motor area, the brainstem and the thalamus (Fig. 5).

Our results confirmed that MVPA is more sensitive than conventional univariate methods in detecting between-condition differences in the fMRI signals. This highlights that the debate on whether the brain responses to transient nociceptive stimuli causing pain detected using fMRI reflect pain-specific neural activity pertains more to the technological than to the physiological domain. Even if the fMRI responses elicited by transient pain should be indistinguishable from the fMRI responses elicited by equally-salient stimuli of other sensory modalities (i.e., stimuli not causing pain), this by no means implies the lack of cortical activities responsible for the painful quality of the percept elicited by the stimulus. Accordingly, classic neurophysiological studies have described nociceptive-specific responses in the supraspinal targets of the spinothalamic pathway. For example, nociceptive-specific neurons have been found in the primary somatosensory cortex (Chen et al. 2009), the ACC (Hutchison et al. 1999) and the insula (Zhang et al. 1999) of animals and humans, although the lack of adequate matching of stimulus saliency between nociceptive and non-nociceptive is also an issue in a number of these previous studies (for an exclusive discussion on this topic, see Mouraux and Iannetti 2018).

It is also interesting to compare the classification patterns identified in the present study with the “Neurologic Pain Signature” described by Wager et al. 2013. When comparing Fig. 5 of the current study and Fig. 1A in Wager et al. 2013 there are both similarities and differences. Both studies found that certain brain regions—dACC/SMA, insula (anterior, mid and posterior), S2 and the thalamus—contained voxels more active during pain versus non-painful sensations (in our study) or during high pain versus low pain (in Wager et al. 2013), again emphasizing the possible role of these regions in pain processing. However, Wager et al. 2013 also identified some negatively-weighted clusters located in the precuneus/PCC, ventral medial prefrontal cortex, occipital gyrus, fusiform and the superior parietal lobule—preferring low pain versus high pain. In contrast, we found very few voxels with consistent negative weights across the three “pain vs. non-pain” classifications. Thus, the contribution of these negatively-weighted regions to pain processing is less certain, and may be dependent on the specific datasets and classification strategies.

It should be noted that, although the successful discrimination of painful versus non-painful stimuli was unlikely consequent to stimulus intensity, saliency or valence (Supplementary Fig. S6), whether the observed spatial pattern of BOLD signal distinguishing pain relates to cortical activity specific for the perception of pain remains an open question. Identifying a specific pattern of neural activity that underlies pain perception is not trivial. To conclude that a neural response is specific for a certain sensation requires comparisons between the neural response elicited by this given sensation and those elicited by all other possible sensations. However, only a limited set of sensations were studied in the present study. In addition, the identified pattern of brain activity elicited by transient laser heat might not generalize to other types of painful percepts, particularly ongoing pain (Baliki et al. 2006). Furthermore, features other than pain perception could have distinguished the painful heat stimuli from the intensity/saliency-matched non-painful tactile, auditory and visual stimuli, and these differences could have contributed to the discrimination between the BOLD responses elicited by painful versus non-painful stimuli. For example, the painful heat stimuli were the only stimuli to have a thermal quality, and were also the only stimuli that engaged the spinothalamic system.

### Brain Responses Distinguishing Stimulus Intensity/Saliency Independently of Sensory Modality

MVPA also distinguished the spatial patterns of the fMRI responses elicited by high-versus low-intensity/saliency stimuli, independently of sensory modality, in both datasets. As aforementioned, stimulus intensity, saliency and valence were highly correlated in our present experimental settings (see Supplementary Fig. S6), and thus we were unable to disentangle the effects caused by these three factors. We also note two additional aspects: (1) classification accuracies in distinguishing high-versus low-intensity/saliency stimuli (Fig. 7 and Supplementary Fig. S17) were generally lower than classification accuracies in distinguishing pain versus touch (Fig. 4 and Supplementary Fig. S9); (2) spatial patterns distinguishing high-versus low-intensity/saliency stimuli obtained separately in different datasets were less generalizable across-datasets (Fig. 7c&d), compared with spatial patterns distinguishing pain versus touch (Fig. 4e&f). There might be several practical reasons that make the classification “high- vs. low-intensity/saliency” more challenging: (1) participants rated stimulus saliency (in Dataset 1) or intensity (in Dataset 2) using different strategies, leading to different ratings in different participants even when the stimuli were perceived similarly; (2) participants had to remember and report at the end of each run of Dataset 1 how they perceived the saliency of 32 stimuli across four modalities, an admittedly suboptimal design that may have reduced the rating accuracy; (3) each stimulus was labeled as “high” or “low” according to median split separately for each dataset; thus, the two classes (“high” and “low”) might not perfectly correspond to each other between the two datasets—increasing the difficulty of the across-datasets classification; (4) in Dataset 2, we collected intensity ratings instead of saliency ratings; although stimulus intensity and saliency were likely to be highly correlated in the design of Experiment 2 (this was also confirmed by the psychophysical experiment, Supplementary Fig. S6), they might not be exactly the same; (5) the signal amplitude in the data had to be normalized to make the two different datasets comparable, thus canceling overall differences in signal amplitude between “high” versus “low” trials.

## Conclusions

Our results are compatible with the view that pain may emerge from neural activity occurring within a distributed large-scale brain network. The finding that information about both stimulus intensity/saliency and pain can be represented by different spatial patterns of activity within the “pain matrix” also demonstrates the complexity of these neural activities, which are likely to subserve multiple functions, some of which may be selective for nociception or pain.

## Supplementary Material

Supplementary Data

## References

[bhz026C1] Afif A, Minotti L, Kahane P, Hoffmann D 2010 Anatomofunctional organization of the insular cortex: a study using intracerebral electrical stimulation in epileptic patients. Epilepsia. 51:2305–2315. 20946128 10.1111/j.1528-1167.2010.02755.x

[bhz026C2] Apkarian AV, Bushnell MC, Treede RD, Zubieta JK 2005 Human brain mechanisms of pain perception and regulation in health and disease. Eur J Pain. 9:463–484. 15979027 10.1016/j.ejpain.2004.11.001

[bhz026C3] Baliki MN, Chialvo DR, Geha PY, Levy RM, Harden RN, Parrish TB, Apkarian AV 2006 Chronic pain and the emotional brain: specific brain activity associated with spontaneous fluctuations of intensity of chronic back pain. J Neurosci. 26:12165–12173. 17122041 10.1523/JNEUROSCI.3576-06.2006 PMC4177069

[bhz026C4] Birn RM, Cornejo MD, Molloy EK, Patriat R, Meier TB, Kirk GR, Nair VA, Meyerand ME, Prabhakaran V 2014 The influence of physiological noise correction on test-retest reliability of resting-state functional connectivity. Brain Connect. 4:511–522. 25112809 10.1089/brain.2014.0284 PMC4146390

[bhz026C5] Boly M, Faymonville ME, Schnakers C, Peigneux P, Lambermont B, Phillips C, Lancellotti P, Luxen A, Lamy M, Moonen G, et al 2008 Perception of pain in the minimally conscious state with PET activation: an observational study. Lancet Neurol. 7:1013–1020. 18835749 10.1016/S1474-4422(08)70219-9

[bhz026C6] Botvinick MM, Cohen JD, Carter CS 2004 Conflict monitoring and anterior cingulate cortex: an update. Trends Cogn Sci. 8:539–546. 15556023 10.1016/j.tics.2004.10.003

[bhz026C7] Chang LJ, Gianaros PJ, Manuck SB, Krishnan A, Wager TD 2015 A sensitive and specific neural signature for picture-induced negative affect. PLoS Biol. 13:e1002180. 26098873 10.1371/journal.pbio.1002180 PMC4476709

[bhz026C8] Chen LM, Friedman RM, Roe AW 2009 Area-specific representation of mechanical nociceptive stimuli within SI cortex of squirrel monkeys. Pain. 141:258–268. 19136211 10.1016/j.pain.2008.11.018 PMC2680084

[bhz026C9] Clark JA, Brown CA, Jones AK, El-Deredy W 2008 Dissociating nociceptive modulation by the duration of pain anticipation from unpredictability in the timing of pain. Clin Neurophysiol. 119:2870–2878. 18980863 10.1016/j.clinph.2008.09.022

[bhz026C10] Corradi-Dell’Acqua C, Tusche A, Vuilleumier P, Singer T 2016 Cross-modal representations of first-hand and vicarious pain, disgust and fairness in insular and cingulate cortex. Nat Commun. 7:10904. 26988654 10.1038/ncomms10904 PMC4802033

[bhz026C11] Craig AD 2009 How do you feel—now? The anterior insula and human awareness. Nat Rev Neurosci. 10:59–70. 19096369 10.1038/nrn2555

[bhz026C12] Dienes Z, Coulton S, Heather N 2018 Using Bayes factors to evaluate evidence for no effect: examples from the SIPS project. Addiction. 113:240–246. 28804980 10.1111/add.14002

[bhz026C13] Downar J, Crawley AP, Mikulis DJ, Davis KD 2000 A multimodal cortical network for the detection of changes in the sensory environment. Nat Neurosci. 3:277–283. 10700261 10.1038/72991

[bhz026C14] Downar J, Crawley AP, Mikulis DJ, Davis KD 2001 The effect of task relevance on the cortical response to changes in visual and auditory stimuli: an event-related fMRI study. Neuroimage. 14:1256–1267. 11707082 10.1006/nimg.2001.0946

[bhz026C15] Downar J, Crawley AP, Mikulis DJ, Davis KD 2002 A cortical network sensitive to stimulus salience in a neutral behavioral context across multiple sensory modalities. J Neurophysiol. 87:615–620. 11784775 10.1152/jn.00636.2001

[bhz026C16] Downar J, Mikulis DJ, Davis KD 2003 Neural correlates of the prolonged salience of painful stimulation. Neuroimage. 20:1540–1551. 14642466 10.1016/s1053-8119(03)00407-5

[bhz026C17] Duerden EG, Albanese MC 2013 Localization of pain-related brain activation: a meta-analysis of neuroimaging data. Hum Brain Mapp. 34:109–149. 22131304 10.1002/hbm.21416 PMC6869965

[bhz026C18] Eickhoff SB, Stephan KE, Mohlberg H, Grefkes C, Fink GR, Amunts K, Zilles K 2005 A new SPM toolbox for combining probabilistic cytoarchitectonic maps and functional imaging data. Neuroimage. 25:1325–1335. 15850749 10.1016/j.neuroimage.2004.12.034

[bhz026C19] Eisenberger NI 2015 Social pain and the brain: controversies, questions, and where to go from here. Annu Rev Psychol. 66:601–629. 25251482 10.1146/annurev-psych-010213-115146

[bhz026C20] Eisenberger NI, Lieberman MD, Williams KD 2003 Does rejection hurt? An FMRI study of social exclusion. Science. 302:290–292. 14551436 10.1126/science.1089134

[bhz026C21] Fazeli S, Buchel C 2018 Pain-related expectation and prediction error signals in the anterior insula are not related to aversiveness. J Neurosci. 38:6461–6474. 29934355 10.1523/JNEUROSCI.0671-18.2018 PMC6705956

[bhz026C22] Friston KJ, Harrison L, Penny W 2003 Dynamic causal modelling. Neuroimage. 19:1273–1302. 12948688 10.1016/s1053-8119(03)00202-7

[bhz026C23] Friston KJ, Holmes AP, Worsley KJ, Poline JB, Frith CD, Frackowiak RSJ 1995 Statistical Parametric Maps in functional imaging: a general linear approach. Hum Brain Mapp. 2:189–210.

[bhz026C24] Garcia-Larrea L, Peyron R 2013 Pain matrices and neuropathic pain matrices: a review. Pain. 154(Suppl 1):S29–S43. 24021862 10.1016/j.pain.2013.09.001

[bhz026C25] Goksan S, Hartley C, Emery F, Cockrill N, Poorun R, Moultrie F, Rogers R, Campbell J, Sanders M, Adams E, et al 2015 fMRI reveals neural activity overlap between adult and infant pain. Elife. 4:e06356 10.7554/eLife.06356 PMC4402596 25895592

[bhz026C26] Hanke M, Halchenko YO, Sederberg PB, Hanson SJ, Haxby JV, Pollmann S 2009 PyMVPA: a python toolbox for multivariate pattern analysis of fMRI data. Neuroinformatics. 7:37–53. 19184561 10.1007/s12021-008-9041-y PMC2664559

[bhz026C27] Hu L, Iannetti GD 2016 Painful issues in pain prediction. Trends Neurosci. 39(4):212–220. 26898163 10.1016/j.tins.2016.01.004

[bhz026C28] Hutchison WD, Davis KD, Lozano AM, Tasker RR, Dostrovsky JO 1999 Pain-related neurons in the human cingulate cortex. Nat Neurosci. 2:403–405. 10321241 10.1038/8065

[bhz026C29] Iannetti GD, Hughes NP, Lee MC, Mouraux A 2008 Determinants of laser-evoked EEG responses: pain perception or stimulus saliency? J Neurophysiol. 100:815–828. 18525021 10.1152/jn.00097.2008 PMC2525705

[bhz026C30] Iannetti GD, Mouraux A 2010 From the neuromatrix to the pain matrix (and back). Exp Brain Res. 205:1–12. 20607220 10.1007/s00221-010-2340-1

[bhz026C31] Iannetti GD, Mouraux A 2015 The ‘pain matrix’: myths and (unpleasant) truths In: Apkarian AV, editor The brain adapting with pain: Contribution of neuroimaging technology to pain mechanisms. Philadelphia: IASP Press.

[bhz026C32] Iannetti GD, Salomons TV, Moayedi M, Mouraux A, Davis KD 2013 Beyond metaphor: contrasting mechanisms of social and physical pain. Trends Cogn Sci. 17:371–378. 23796880 10.1016/j.tics.2013.06.002

[bhz026C33] Isnard J, Magnin M, Jung J, Mauguiere F, Garcia-Larrea L 2011 Does the insula tell our brain that we are in pain? Pain. 152:946–951. 21277680 10.1016/j.pain.2010.12.025

[bhz026C34] Jones TB, Bandettini PA, Birn RM 2008 Integration of motion correction and physiological noise regression in fMRI. Neuroimage. 42:582–590. 18583155 10.1016/j.neuroimage.2008.05.019 PMC2833099

[bhz026C35] Kragel PA, Kano M, Van Oudenhove L, Ly HG, Dupont P, Rubio A, Delon-Martin C, Bonaz BL, Manuck SB, Gianaros PJ, et al 2018 Generalizable representations of pain, cognitive control, and negative emotion in medial frontal cortex. Nat Neurosci. 21:283–289. 29292378 10.1038/s41593-017-0051-7 PMC5801068

[bhz026C36] Krishnan A, Woo CW, Chang LJ, Ruzic L, Gu X, Lopez-Sola M, Jackson PL, Pujol J, Fan J, Wager TD 2016 Somatic and vicarious pain are represented by dissociable multivariate brain patterns. Elife. 5. 10.7554/eLife.15166 PMC4907690 27296895

[bhz026C37] Kross E, Berman MG, Mischel W, Smith EE, Wager TD 2011 Social rejection shares somatosensory representations with physical pain. Proc Natl Acad Sci USA. 108:6270–6275. 21444827 10.1073/pnas.1102693108 PMC3076808

[bhz026C38] Kucyi A, Davis KD 2015 The dynamic pain connectome. Trends Neurosci. 38:86–95. 25541287 10.1016/j.tins.2014.11.006

[bhz026C39] Legrain V, Iannetti GD, Plaghki L, Mouraux A 2011 The pain matrix reloaded: a salience detection system for the body. Prog Neurobiol. 93:111–124. 21040755 10.1016/j.pneurobio.2010.10.005

[bhz026C40] Liang M, Mouraux A, Hu L, Iannetti GD 2013 Primary sensory cortices contain distinguishable spatial patterns of activity for each sense. Nat Commun. 4:1979. 23752667 10.1038/ncomms2979 PMC3709474

[bhz026C41] Lieberman MD, Eisenberger NI 2015 The dorsal anterior cingulate cortex is selective for pain: Results from large-scale reverse inference. Proc Natl Acad Sci USA. 112(49):15250–15255. 26582792 10.1073/pnas.1515083112 PMC4679028

[bhz026C42] Lorenz J, Minoshima S, Casey KL 2003 Keeping pain out of mind: the role of the dorsolateral prefrontal cortex in pain modulation. Brain. 126:1079–1091. 12690048 10.1093/brain/awg102

[bhz026C43] Macdonald G, Leary MR 2005 Why does social exclusion hurt? The relationship between social and physical pain. Psychol Bull. 131:202–223. 15740417 10.1037/0033-2909.131.2.202

[bhz026C44] Melzack R 1989 Labat lecture. Phantom limbs. Reg Anesth. 14:208–211. 2486645

[bhz026C45] Melzack R 2001 Pain and the neuromatrix in the brain. J Dent Educ. 65:1378–1382. 11780656

[bhz026C46] Menon V, Uddin LQ 2010 Saliency, switching, attention and control: a network model of insula function. Brain Struct Funct. 214:655–667. 20512370 10.1007/s00429-010-0262-0 PMC2899886

[bhz026C47] Morey RD, Romeijn J-W, Rouder JN 2016 The philosophy of Bayes factors and the quantification of statistical evidence. J Math Psychol. 72:6–18.

[bhz026C48] Mouraux A, Diukova A, Lee MC, Wise RG, Iannetti GD 2011 A multisensory investigation of the functional significance of the “pain matrix”. Neuroimage. 54:2237–2249. 20932917 10.1016/j.neuroimage.2010.09.084

[bhz026C49] Mouraux A, Iannetti GD 2009 Nociceptive laser-evoked brain potentials do not reflect nociceptive-specific neural activity. J Neurophysiol. 101:3258–3269. 19339457 10.1152/jn.91181.2008

[bhz026C73] Mouraux A, Iannetti GD 2018 The search for pain biomarkers in the human brain. Brain. 141:3290–3307. 30462175 10.1093/brain/awy281 PMC6262221

[bhz026C50] Mur M, Bandettini PA, Kriegeskorte N 2009 Revealing representational content with pattern-information fMRI—an introductory guide. Soc Cogn Affect Neurosci. 4:101–109. 19151374 10.1093/scan/nsn044 PMC2656880

[bhz026C51] Ostrowsky K, Magnin M, Ryvlin P, Isnard J, Guenot M, Mauguiere F 2002 Representation of pain and somatic sensation in the human insula: a study of responses to direct electrical cortical stimulation. Cereb Cortex. 12:376–385. 11884353 10.1093/cercor/12.4.376

[bhz026C52] Penfield W, Boldrey E 1937 Somatic motor and sensory representation in the cerebral cortex of main as studied by electrical stimulation. Brain. 60:383–443.

[bhz026C53] Pereira F, Mitchell T, Botvinick M 2009 Machine learning classifiers and fMRI: a tutorial overview. Neuroimage. 45:S199–S209. 19070668 10.1016/j.neuroimage.2008.11.007 PMC2892746

[bhz026C54] Peyron R, Frot M, Schneider F, Garcia-Larrea L, Mertens P, Barral FG, Sindou M, Laurent B, Mauguière F 2002 Role of operculoinsular cortices in human pain processing: converging evidence from PET, fMRI, dipole modeling, and intracerebral recordings of evoked potentials. Neuroimage. 17:1336–1346. 12414273 10.1006/nimg.2002.1315

[bhz026C55] Protzner AB, McIntosh AR 2006 Testing effective connectivity changes with structural equation modeling: what does a bad model tell us? Hum Brain Mapp. 27:935–947. 16929548 10.1002/hbm.20233 PMC6871338

[bhz026C56] Renier LA, Anurova I, De Volder AG, Carlson S, VanMeter J, Rauschecker JP 2009 Multisensory integration of sounds and vibrotactile stimuli in processing streams for “what” and “where”. J Neurosci. 29:10950–10960. 19726653 10.1523/JNEUROSCI.0910-09.2009 PMC3343457

[bhz026C57] Rouder JN, Speckman PL, Sun D, Morey RD, Iverson G 2009 Bayesian t tests for accepting and rejecting the null hypothesis. Psychon Bull Rev. 16:225–237. 19293088 10.3758/PBR.16.2.225

[bhz026C58] Salomons TV, Iannetti GD, Liang M, Wood JN 2016 The “Pain Matrix” in Pain-Free Individuals. JAMA Neurol. 73(6):755–756. 27111250 10.1001/jamaneurol.2016.0653

[bhz026C59] Segerdahl AR, Mezue M, Okell TW, Farrar JT, Tracey I 2015 The dorsal posterior insula subserves a fundamental role in human pain. Nat Neurosci. 18(4):499–500. 25751532 10.1038/nn.3969 PMC6783299

[bhz026C60] Shackman AJ, Salomons TV, Slagter HA, Fox AS, Winter JJ, Davidson RJ 2011 The integration of negative affect, pain and cognitive control in the cingulate cortex. Nat Rev Neurosci. 12:154–167. 21331082 10.1038/nrn2994 PMC3044650

[bhz026C61] Sharvit G, Corradi-Dell’Acqua C, Vuilleumier P 2018 Modality-specific effects of aversive expectancy in the anterior insula and medial prefrontal cortex. Pain. 159:1529–1542. 29613910 10.1097/j.pain.0000000000001237

[bhz026C62] Spence C 2009 Explaining the Colavita visual dominance effect. Prog Brain Res. 176:245–258. 19733761 10.1016/S0079-6123(09)17615-X

[bhz026C63] Tracey I, Mantyh PW 2007 The cerebral signature for pain perception and its modulation. Neuron. 55:377–391. 17678852 10.1016/j.neuron.2007.07.012

[bhz026C64] Treede RD, Kenshalo DR, Gracely RH, Jones AK 1999 The cortical representation of pain. Pain. 79:105–111. 10068155 10.1016/s0304-3959(98)00184-5

[bhz026C65] Treede RD, Lorenz J, Baumgartner U 2003 Clinical usefulness of laser-evoked potentials. Neurophysiol Clin. 33:303–314. 14678844 10.1016/j.neucli.2003.10.009

[bhz026C66] Tzourio-Mazoyer N, Landeau B, Papathanassiou D, Crivello F, Etard O, Delcroix N, Mazoyer B, Joliot M 2002 Automated anatomical labeling of activations in SPM using a macroscopic anatomical parcellation of the MNI MRI single-subject brain. Neuroimage. 15:273–289. 11771995 10.1006/nimg.2001.0978

[bhz026C67] Vogt BA, Finch DM, Olson CR 1992 Functional heterogeneity in cingulate cortex: the anterior executive and posterior evaluative regions. Cereb Cortex. 2:435–443. 1477524 10.1093/cercor/2.6.435-a

[bhz026C68] Wager TD, Atlas LY, Botvinick MM, Chang LJ, Coghill RC, Davis KD, Iannetti GD, Poldrack RA, Shackman AJ, Yarkoni T 2016 Pain in the ACC? Proc Natl Acad Sci USA. 113:E2474–E2475. 27095849 10.1073/pnas.1600282113 PMC4983860

[bhz026C69] Wager TD, Atlas LY, Lindquist MA, Roy M, Woo CW, Kross E 2013 An fMRI-based neurologic signature of physical pain. N Engl J Med. 368:1388–1397. 23574118 10.1056/NEJMoa1204471 PMC3691100

[bhz026C70] Woo CW, Koban L, Kross E, Lindquist MA, Banich MT, Ruzic L, Andrews-Hanna JR, Wager TD 2014 Separate neural representations for physical pain and social rejection. Nat Commun. 5:5380. 25400102 10.1038/ncomms6380 PMC4285151

[bhz026C71] Yarkoni T, Poldrack RA, Nichols TE, Van Essen DC, Wager TD 2011 Large-scale automated synthesis of human functional neuroimaging data. Nat Methods. 8:665–670. 21706013 10.1038/nmeth.1635 PMC3146590

[bhz026C72] Zhang ZH, Dougherty PM, Oppenheimer SM 1999 Monkey insular cortex neurons respond to baroreceptive and somatosensory convergent inputs. Neuroscience. 94:351–360. 10579199 10.1016/s0306-4522(99)00339-5

