# Supplementary material for: Spatial Patterns of Brain Activity Preferentially Reflecting Transient Pain and Stimulus Intensity
Source: Cereb Cortex. 2019 Mar 7;29(5):2211–27. doi: 10.1093/cercor/bhz026 (PMC6458907; doi:10.1093/cercor/bhz026)
Supplement: Supplementary Data [file bhz026_supplemental_20190129.docx]

Supplemental Information

for

Spatial patterns of brain activity preferentially reflecting transient pain and stimulus intensity

M Liang, Q Su, A Mouraux, GD Iannetti

**Supplemental Methods and Results**

***Psychophysical examination of the relationship between stimulus intensity, saliency and valence***

The aim of this additional psychophysical experiment was to examine the relationship between the perceived intensity, the saliency and the valence of the stimuli used in the main fMRI experiments. Twenty-two healthy participants (17 females, aged 21-28 years) took part in this experiment after providing written informed consent. The local Ethics Committee approved the experimental procedures.

***Experimental design.*** Each participant received stimuli identical to those used in Datasets 1 and 2: painful stimuli were infrared laser pulses delivered to the foot dorsum and tactile stimuli were transcutaneous electrical stimulation of the superficial peroneal nerve at the level of the ankle. Three stimulus intensities were used: 2.75 J, 3.5 J, 4.25 J for laser stimuli and 3 mA, 6 mA, 9 mA for electrical stimuli. The experiment consisted of two sessions. Each session consisted of two blocks in which only painful stimuli were delivered, and two blocks in which only tactile stimuli were delivered. Each block consisted of six trials. Thus, in each session a total of 12 painful stimuli and 12 tactile stimuli were delivered. At the beginning of each trial a fixation cross was presented at the centre of a screen for 15 s, and meantime the stimulus was delivered with a random onset between 2 and 12 s. After the first 15 s, the fixation cross disappeared, and participants were prompted to provide three ratings for the stimulus received using three visual analogue scales (VAS): intensity, saliency and valence. Intensity and saliency ratings were collected using the same VAS used in Datasets 1 and 2. Valence ratings were collected using a validated VAS ranging between 1 and 9, where 5 indicates neutral valence, <5 indicates negative valence and >5 indicates positive valence ([Chikazoe et al. 2014](#_ENREF_1); [Nummenmaa et al. 2014](#_ENREF_5); [Vigliocco et al. 2014](#_ENREF_7); [Kim et al. 2017](#_ENREF_3)). Participants provided VAS ratings by pressing two buttons with their right index or middle finger. The order of ratings were pseudorandomized. Each VAS scale was presented on the screen for 8 s, resulting in a total duration of 39 s (15 + 8×3 s) for each trial. Therefore, each session lasted about 16 min.

***Data analysis.*** For each participant, we first selected a subset of painful and tactile stimuli whose single-trial intensity ratings were matched between the two conditions. This matching was obtained with the same procedure used in Dataset 2. Second, single-stimulus saliency (or valence) ratings of each participant were averaged across the intensity-matched stimuli, separately for the pain and touch conditions. Third, the average saliency (or valence) ratings were compared between the pain and touch conditions using a two-sample T test as well as a model selection method based on Bayes Factor ([Rouder et al. 2009](#_ENREF_6); [Morey et al. 2016](#_ENREF_4); [Dienes et al. 2018](#_ENREF_2)). Finally, a Pearson’s correlation analysis was performed to test whether saliency (or valence) ratings were correlated with intensity ratings.

***Univariate GLM analyses of Dataset 1 and Dataset 2 processed using the same pipeline.***

As Datasets 1 and 2 were collected and analysed for different purposes, their processing pipelines were not identical. Therefore, to test whether the GLM results depended on the actual processing pipeline, we performed a second univariate GLM analysis in which both datasets were processed using the same pipeline and software.

***Data analysis.*** Preprocessing steps included: motion correction, normalization to standard MNI space, re-sampling to 3x3x3 mm^3^ voxel size, spatial smoothing with a Gaussian kernel with FWHM of 8 mm, and high-pass temporal filtering (1/128 Hz cutoff). The first and second level GLM analyses were identical to those described in the main text. Statistical significance was determined using both voxel-based FWE correction (P<0.05) and cluster-based FWE correction (P<0.05, cluster defining threshold P<0.001).

***Results.*** Results are shown in Supplemental Figure S7. When assessed using the most severe voxel-based FWE correction, results of both Dataset 1 and Dataset 2 were very similar to those obtained using the original processing pipeline: for Dataset 1, there was no difference in each of the three pain vs no-pain comparisons (i.e., ‘pan vs. touch’, ‘pain vs. audition’ and ‘pain vs. vision’); for Dataset 2, only the left anterior insula and the bilateral frontal operculum showed significantly higher activation for pain than for touch. However, when assessed using the less severe cluster-based FWE correction, the results obtained from both Dataset 1 and Dataset 2 changed to some extent: for Dataset 1, several clusters in the bilateral thalamus and bilateral insula showed higher activation for pain than for touch; in contrast, for Dataset 2, only the right frontal operculum showed higher activation for pain than for touch.


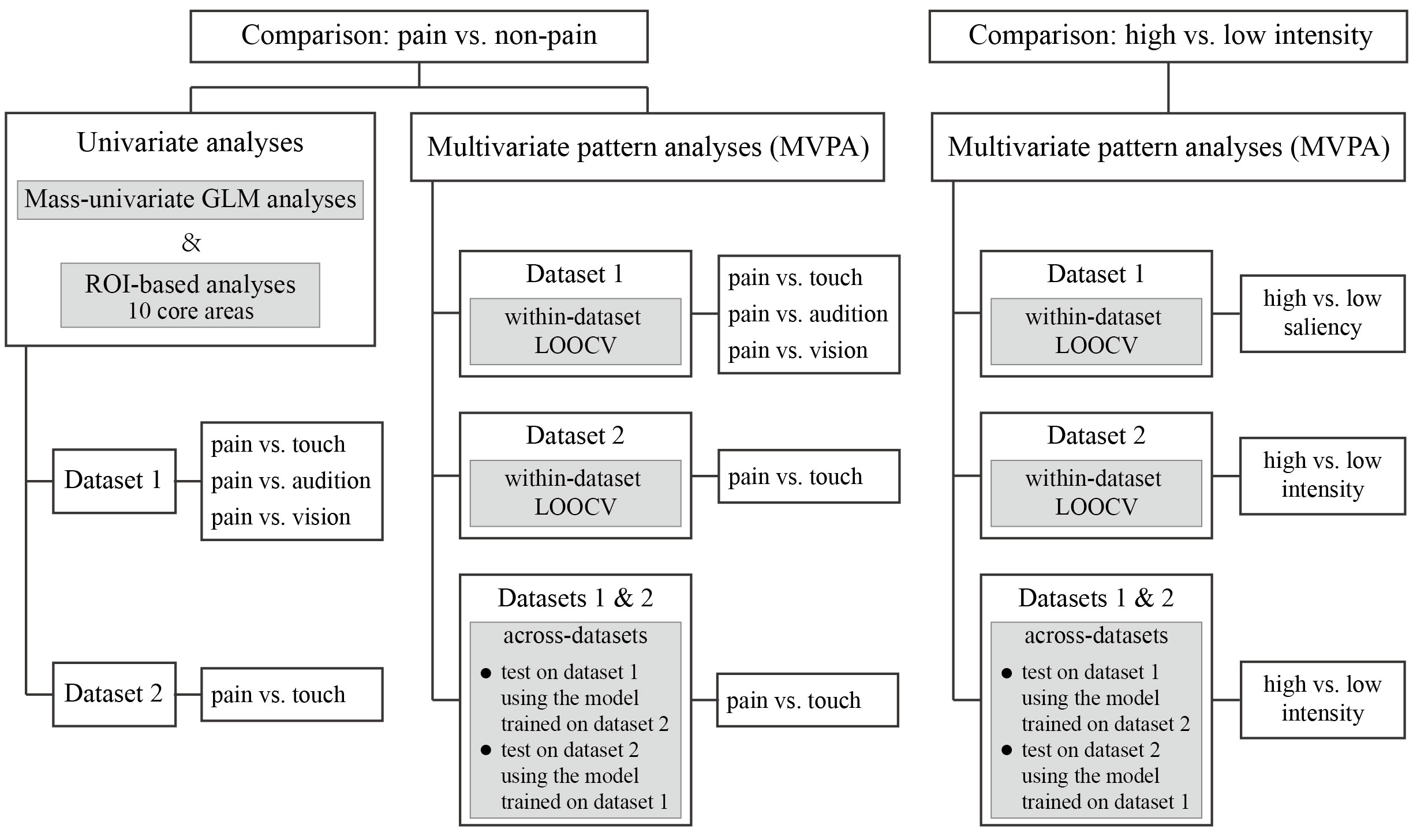


**Figure S1.** Overview of the different analyses. Two comparisons (‘pain vs. non-pain’ and ‘high vs. low intensity/saliency’) were performed in two datasets (Dataset 1 and Dataset 2). After matching stimulus intensity/saliency between pain and non-pain conditions, the fMRI responses elicited by different stimuli were compared using two types of univariate analyses (conventional voxel-wise GLM analysis and ROI-based analysis), as well as using multivariate pattern analysis (MVPA). Univariate analyses were performed separately on each of the two datasets. In contrast, MVPA was performed within Dataset 1, within Dataset 2, and across the two datasets, using a leave-one-out cross validation (LOOCV) procedure. For the comparison of ‘high vs. low intensity/saliency’, stimuli were divided into two groups according to the subjective saliency ratings (Dataset 1) or the subjective intensity ratings (Dataset 2), and the corresponding fMRI responses were compared using MVPA.


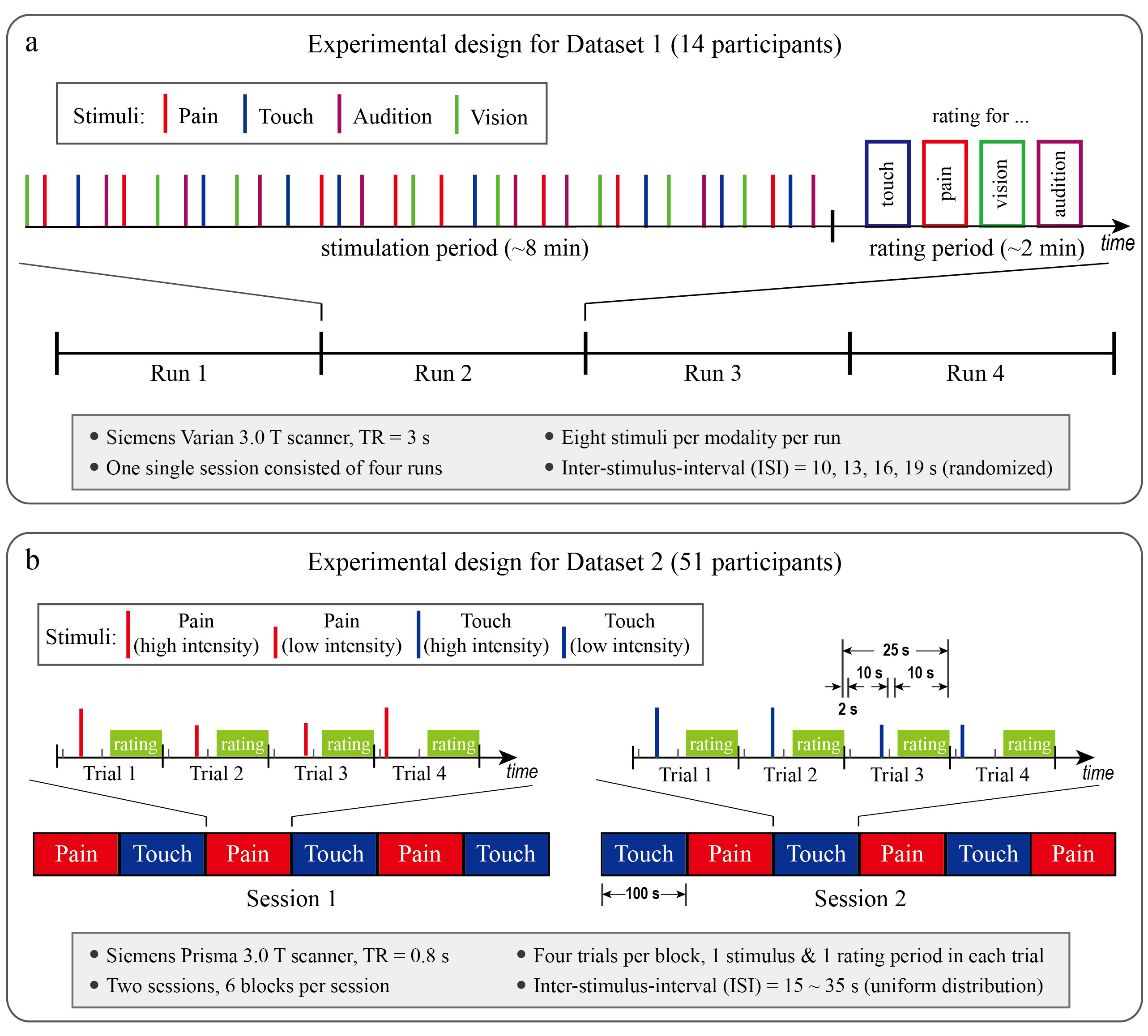


**Figure S2.** Experimental designs used to collect Dataset 1 (**a**) and Dataset 2 (**b**).


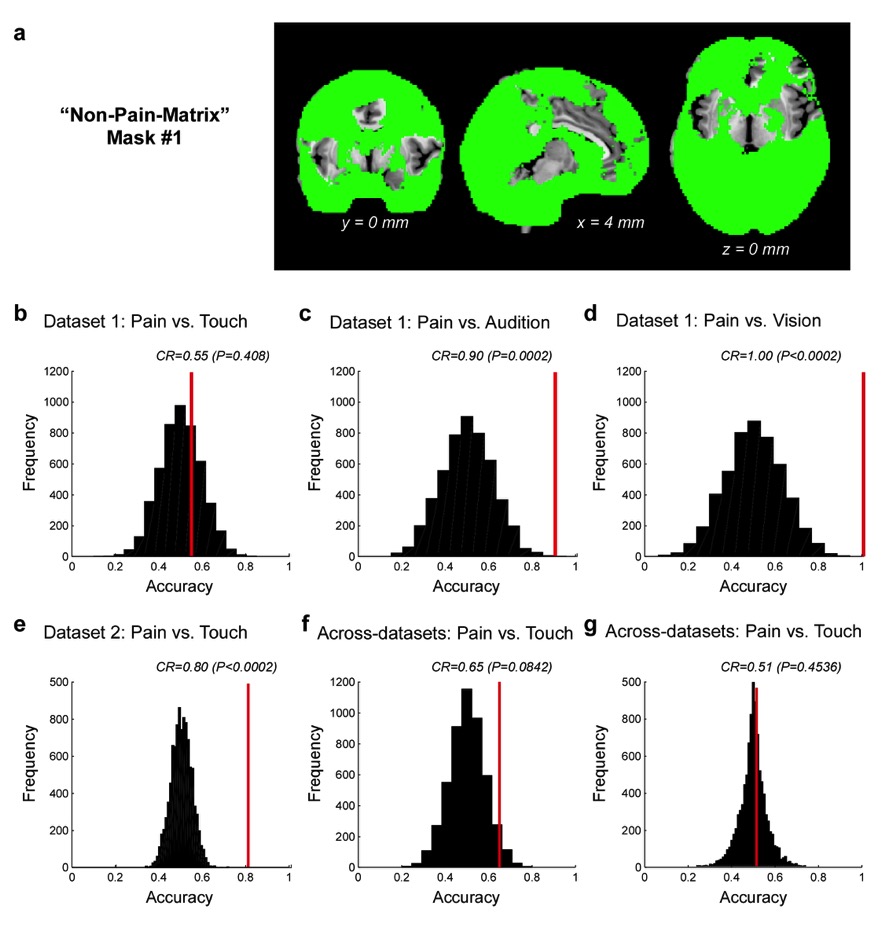


**Figure S3.** Panel **a:** whole “non-pain-matrix” mask created by subtracting the voxels of the "pain matrix" mask from the whole brain mask. Panels **b-d:** results obtained from normalized Dataset 1 for the three classification tasks, respectively. Panel **e:** result obtained from normalized Dataset 2 for the ‘pain vs. touch’ classification task. Panel **f:** result obtained using Dataset 2 as training dataset and Dataset 1 as test dataset. Panel **g:** result obtained using Dataset 1 as training dataset and Dataset 2 as test dataset. Classification accuracies (correct rate, CR) are indicated by red vertical lines and corresponding null distributions (obtained from 5,000 permutations) are indicated by black bell shapes. P-values were calculated as the proportion of how many (out of 5,000) permutations generated accuracy greater than or equal to the actual classification accuracy.


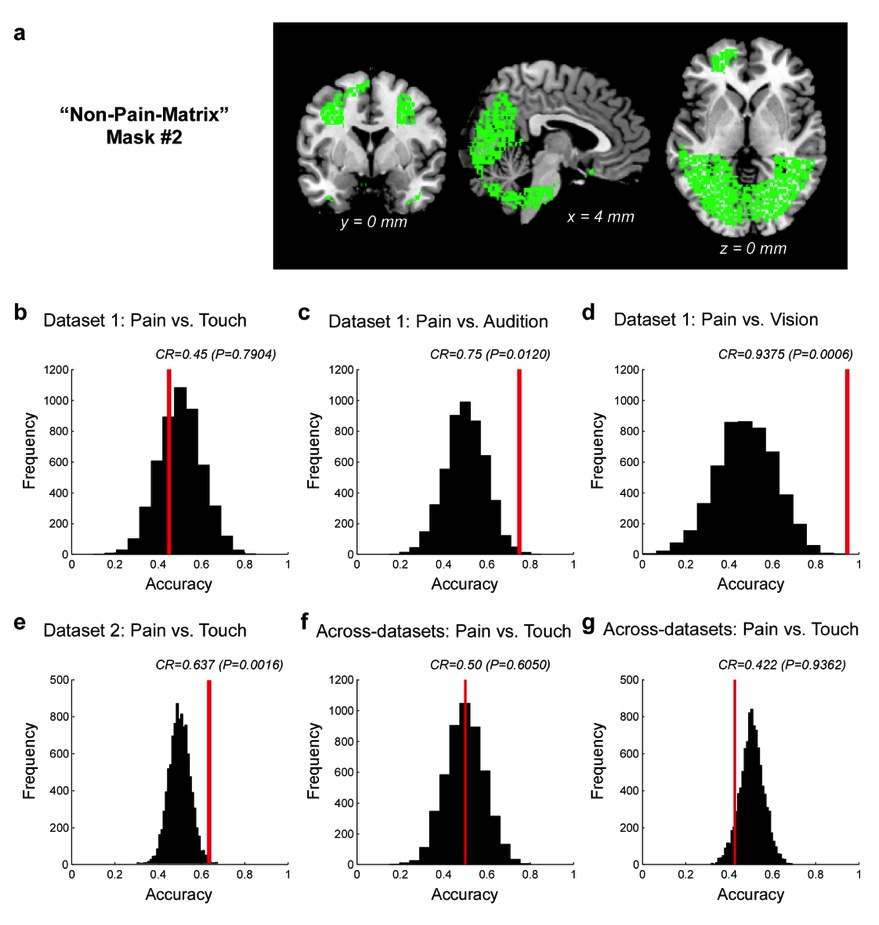


**Figure S4.** Panel **a:** the eroded “non-pain-matrix” mask was generated by eroding the first “non-pain-matrix” mask and then randomly selecting a number of voxels equal to the “pain matrix” mask. Panels **b-d:** results obtained from normalized Dataset 1 for the three classification tasks, respectively. Panel **e**: result obtained from normalized Dataset 2 for the ‘pain vs. touch’ classification task. Panel **f**: result obtained using Dataset 2 as training dataset and Dataset 1 as test dataset. Panel **g**: result obtained using Dataset 1 as training dataset and Dataset 2 as test dataset. Classification accuracies (correct rate, CR) are indicated by red vertical lines and corresponding null distributions (obtained from 5,000 permutations) are indicated by black bell shapes. P-values were calculated as the proportion of how many (out of 5,000) permutations generated accuracy greater than or equal to the actual classification accuracy.


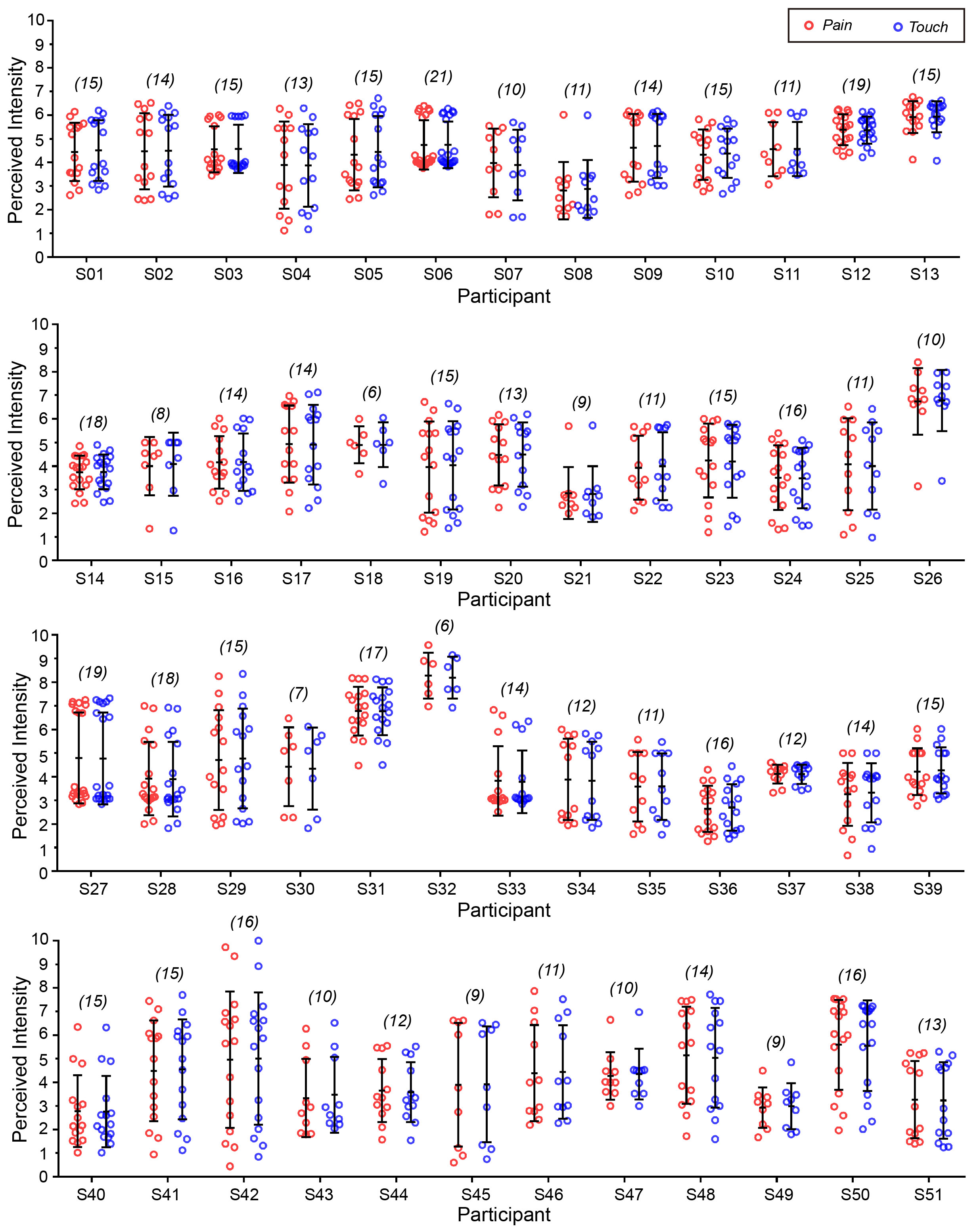

**Figure S5.** Scatter diagrams of subjective intensity ratings of all single stimuli with intensity matched across the pain and touch conditions, for each participant. The number of pairs of pain and touch stimuli with matched intensity is shown in parenthesis. Red indicates painful stimuli and blue indicates tactile stimuli.


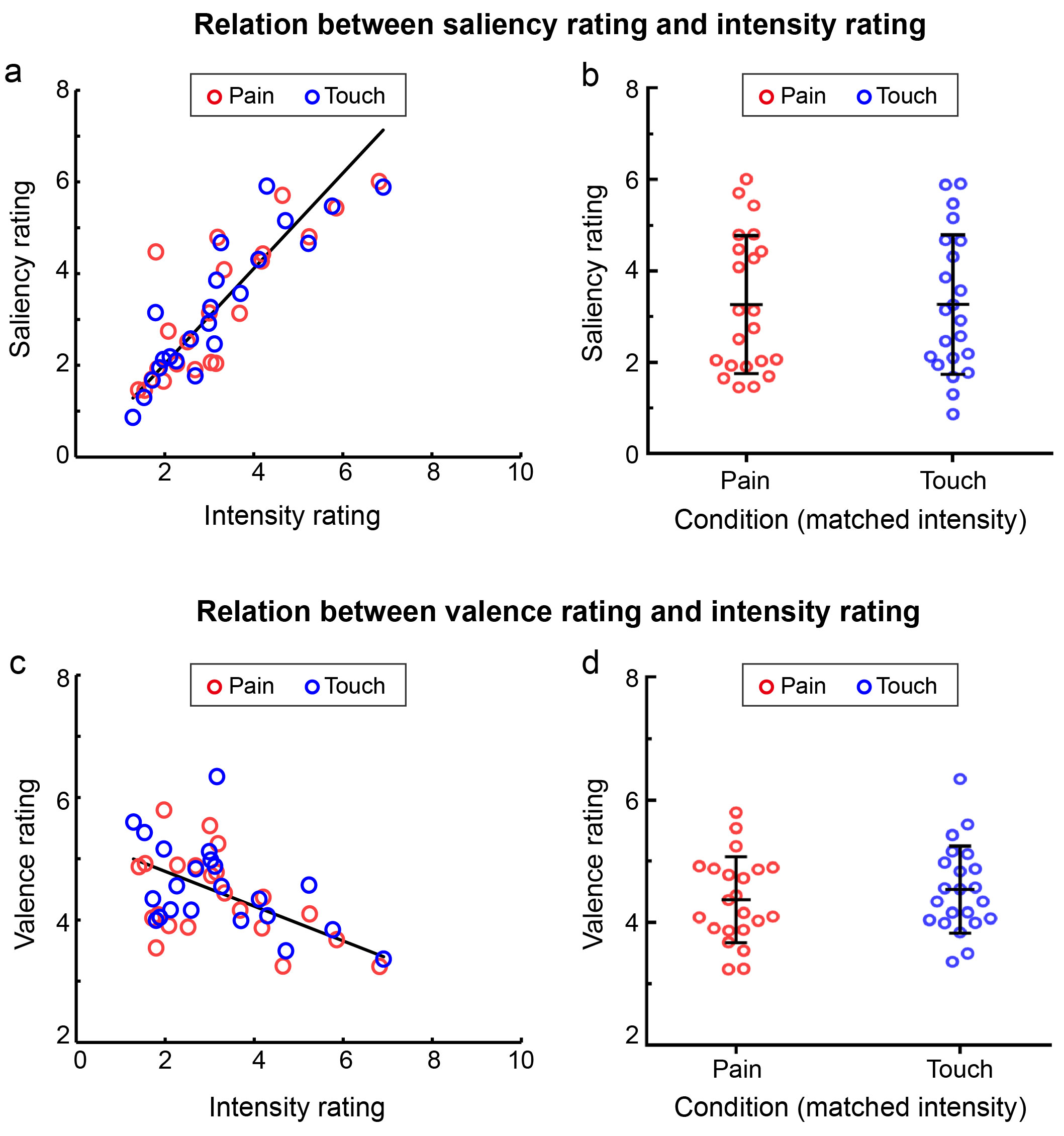


**Figure S6.** Relationship between intensity, saliency and valence ratings of painful and tactile stimuli. We observed strong evidence that saliency and intensity ratings were positively correlated (R =0.86, P =6.92×10^-14^; panel **a**). Also, saliency ratings were similar for intensity-matched painful and tactile stimuli (T =-0.0055, P =0.9956, BF_01_ =4.49; panel **b**). In contrast, we observed strong evidence that valence and intensity ratings were negatively correlated (R =-0.49, P =7.66×10^-4^; panel **c**; note that the lower the rating, the higher the negative valence). Valence ratings were similar for intensity-matched painful and tactile stimuli (T =-0.77, P =0.44, BF_01_ =3.44; panel d). BF_01_, Bayes factor.


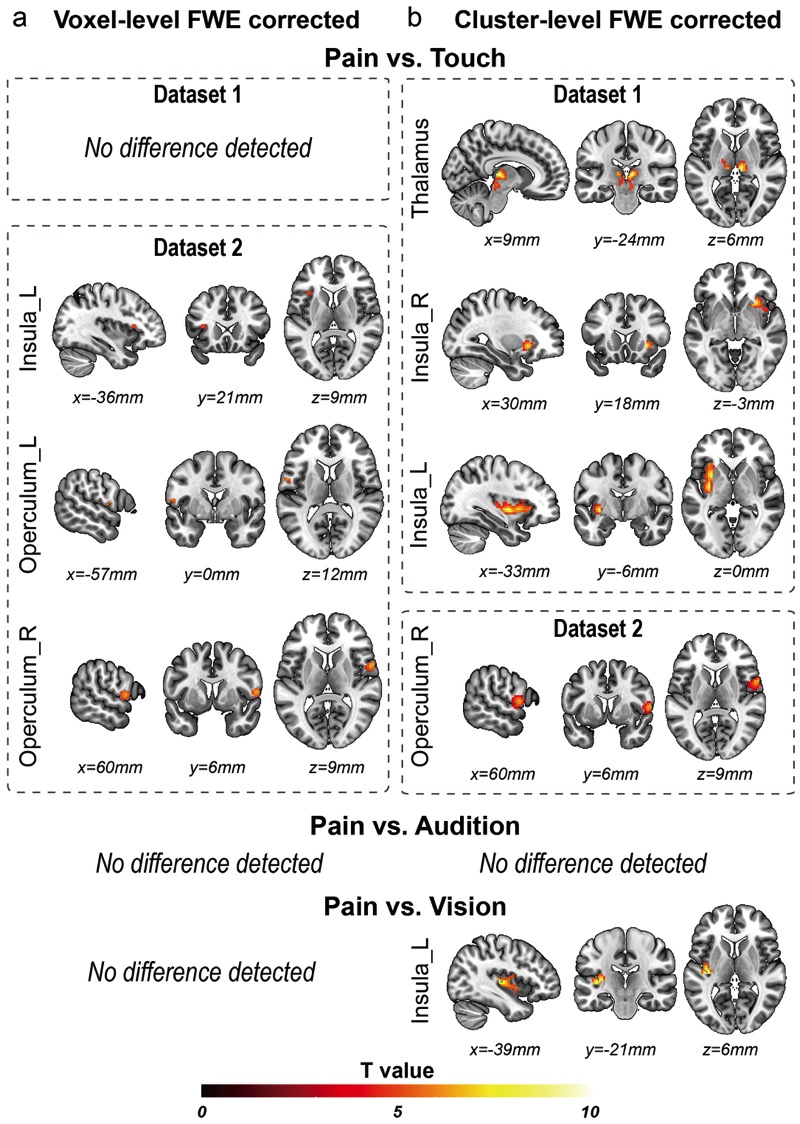


**Figure S7.** Univariate GLM results obtained using Datasets 1 and 2 processed using the same pipeline (see Supplemental Methods). Panels **a** and **b:** results obtained using voxel-based and cluster-based FWE correction (P<0.05 corrected) in Datasets 1 and 2, respectively.


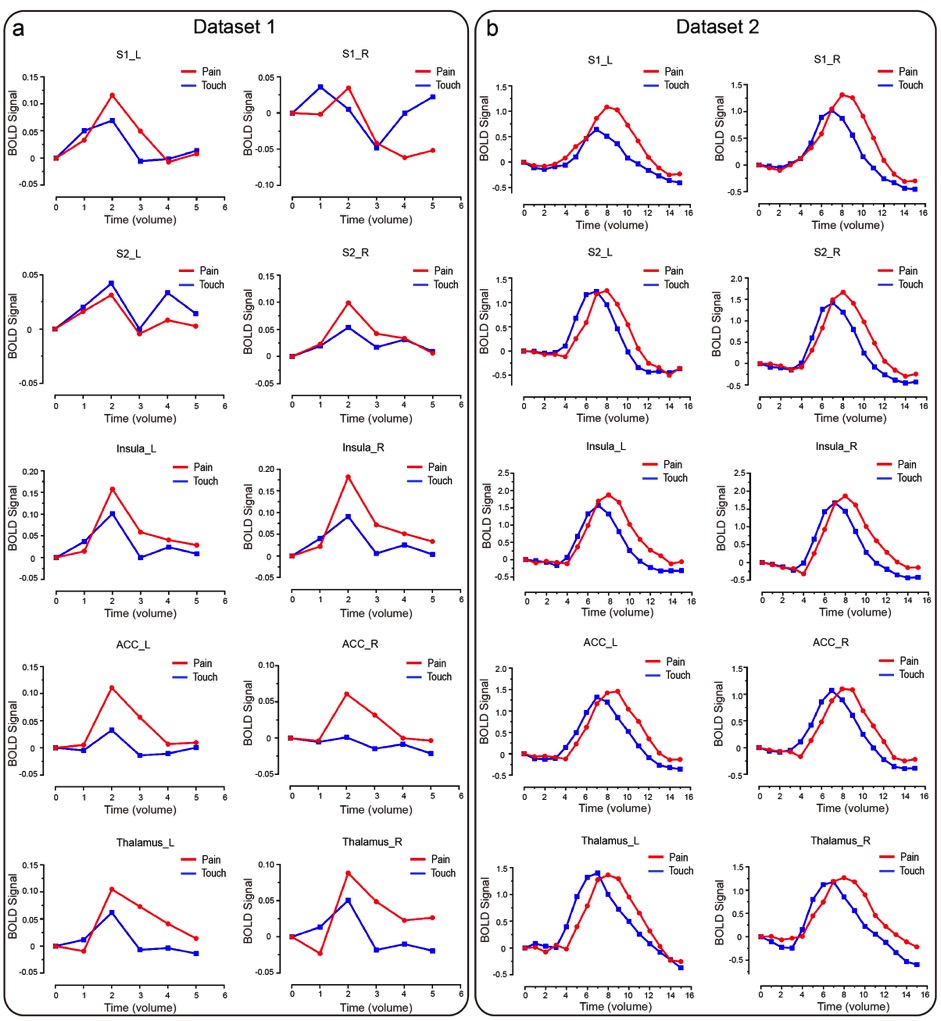


**Figure S8.** Time courses of raw fMRI responses elicited by painful stimuli (red) and tactile stimuli (blue) in the ten ROIs obtained from Dataset 1 (**a**) and Dataset 2 (**b**). The x-axis indicates the volume (i.e., TR) after stimulus onset: e.g., the number ‘2’ of the x-axis means the 2^nd^ volume after stimulus onset. Note that TR=3 s in Dataset 1 and TR=0.8 s in Dataset 2.


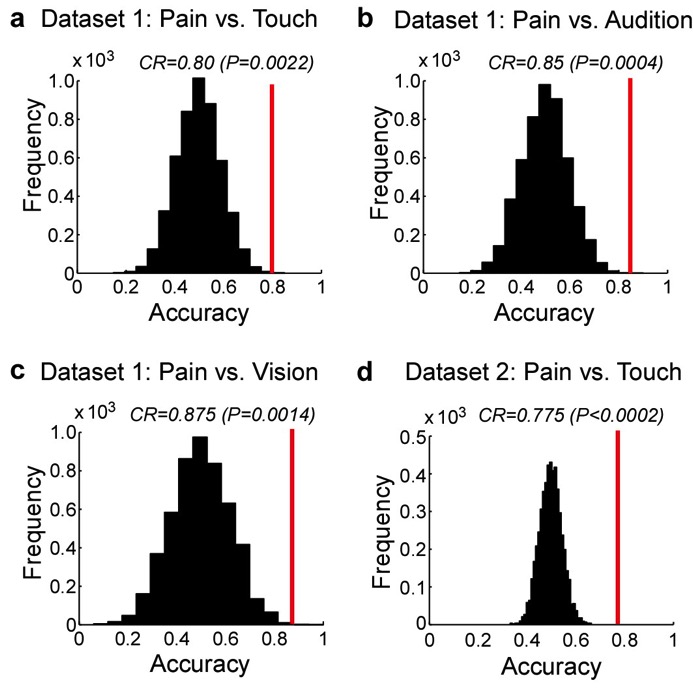


**Figure S9.** Classification accuracies (correct rates, CR) of ‘pain vs. non pain’ classifications performed on non-normalized data, along with the corresponding null distributions, using Dataset 1 (**a**-**c**) and Dataset 2 (**d**). Panels **a**-**c**: results obtained from Dataset 1 for the three classification tasks, respectively. Panel **d**: result obtained from Dataset 2 for the ‘pain vs. touch’ classification task. Classification accuracies are indicated by red vertical lines and corresponding null distributions (obtained from 5,000 permutations) are indicated by black bell shapes. P-values were calculated as the proportion of how many (out of 5,000) permutations generated accuracy greater than or equal to the actual classification accuracy.


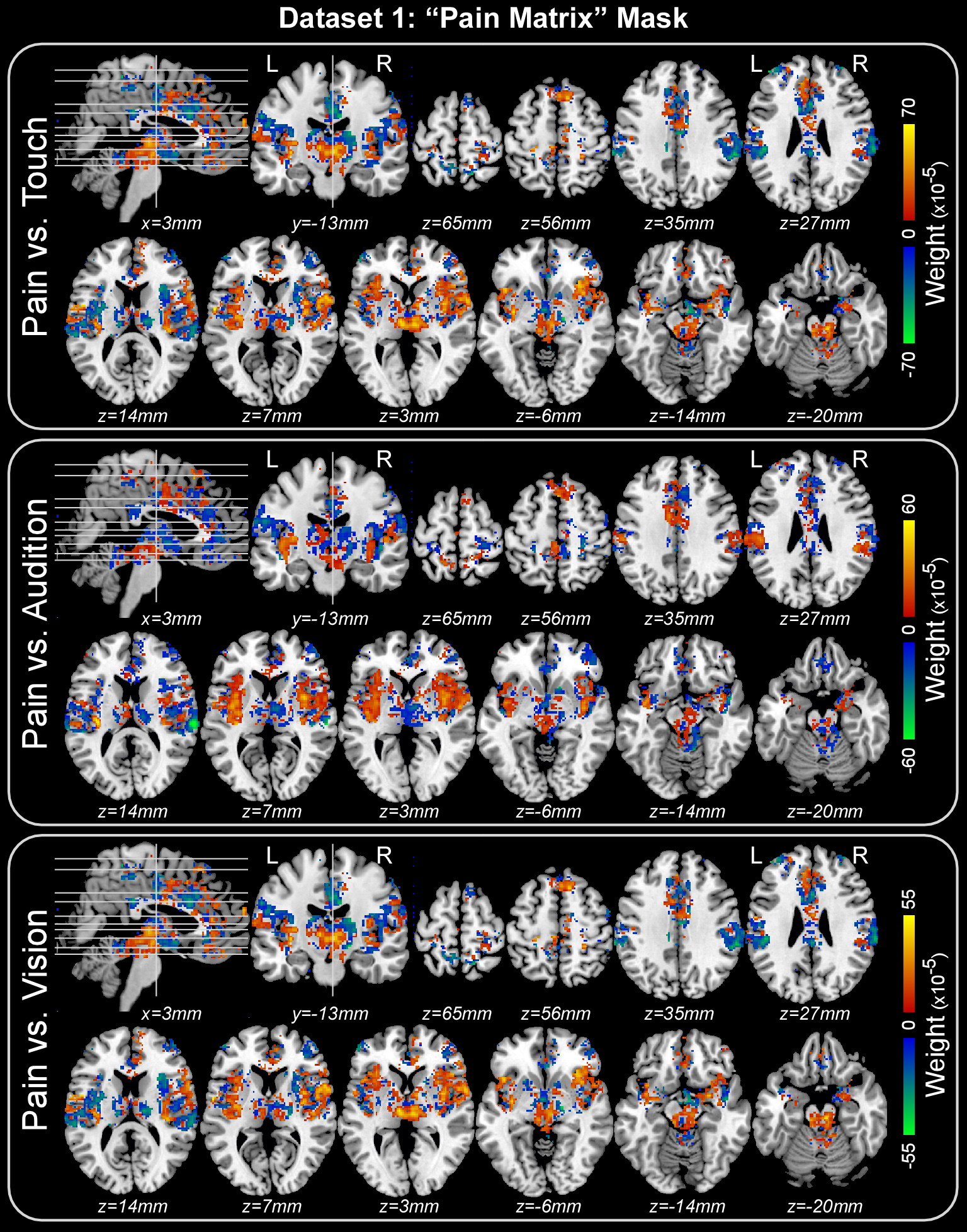


**Figure S10.** Dataset 1. Sensitivity maps of the classifications pain vs each of the other sensory modalities obtained using the normalised signal of the “pain matrix” mask. The maps show the SVM weight of each voxel. Only voxels with a consistent sign of weight across all cross validations are shown. A positive sign implies that the voxel had higher BOLD signal during pain (red-yellow), whereas a negative sign implies that the voxel had higher BOLD signal during the non-painful stimulation (blue-green) in a given classification task.


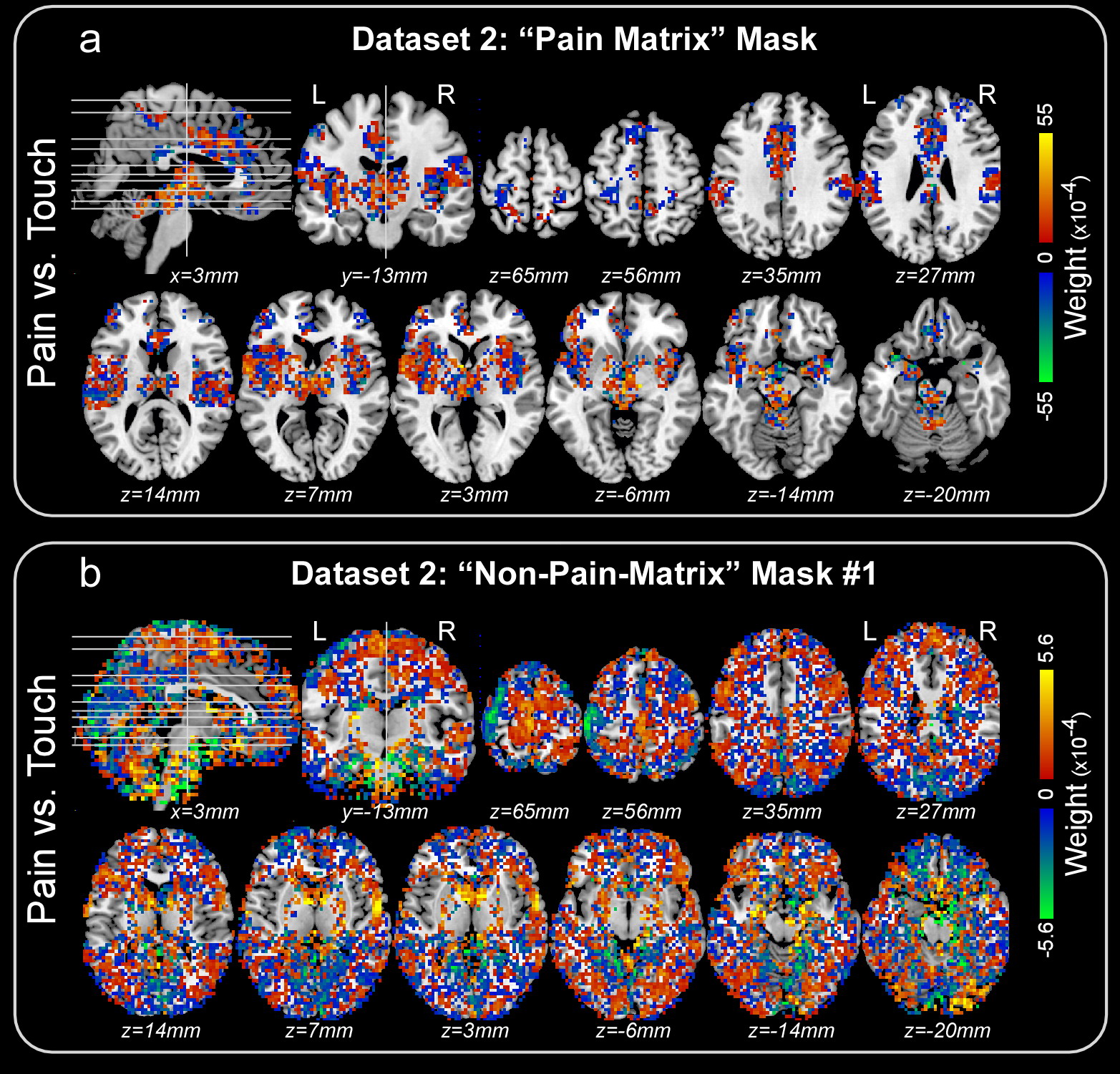


**Figure S11.** Sensitivity maps of the classification “pain vs. touch” obtained using the “pain matrix” mask (**a**) and the first “non-pain matrix” mask (**b**) of Dataset 2, when the signal of the “pain matrix” mask of Dataset 2 was normalised. These maps show the SVM weight of each voxel. Only voxels with a consistent sign of weight across all cross validations are shown. A positive sign implies that the voxel had higher BOLD signal during pain (red-yellow), whereas a negative sign implies that the voxel had higher BOLD signal during touch (blue-green).


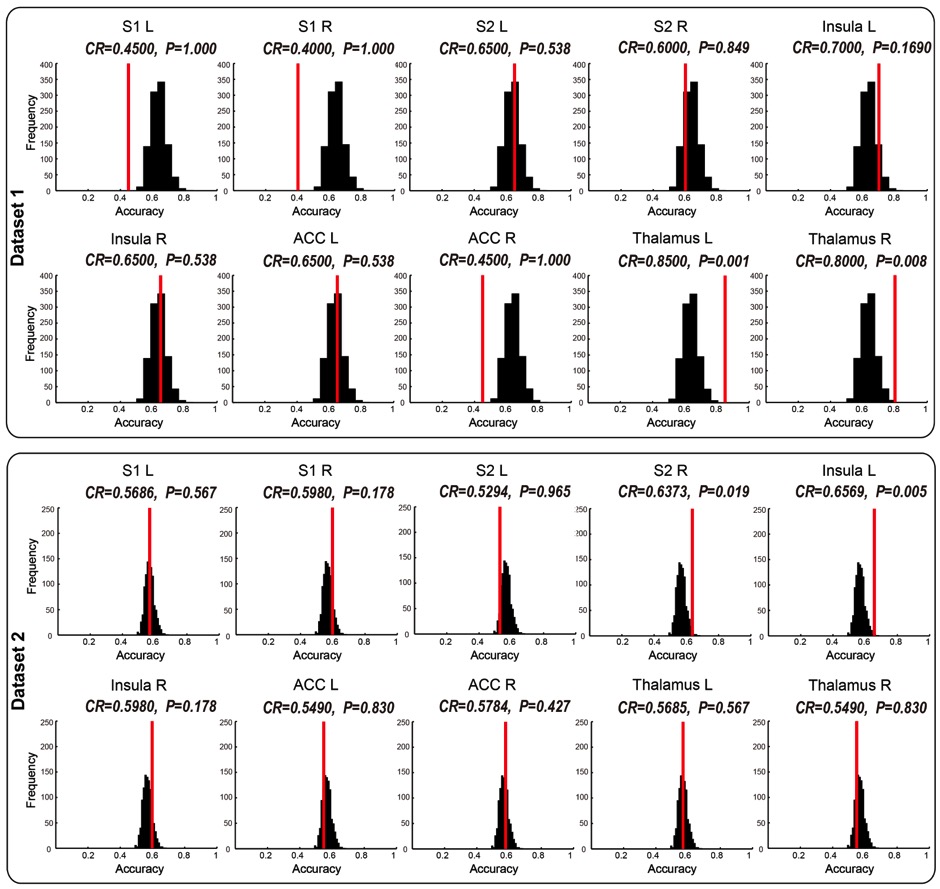


**Figure S12.** Pain vs. touch within-dataset classification accuracies obtained using normalised fMRI signals (upper panel: within Dataset 1; lower panel: within Dataset 2). Classification accuracies (correct rate, CR) are indicated by red vertical lines. The statistical significance was determined by 1,000 permutations and the resulting p-value was corrected for the 10 ROIs examined (FWE corrected), as follows. We took the maximal accuracy of the 10 ROIs for each permutation and then built a null distribution using the 1,000 maximal accuracies (i.e., the black bell shapes). The bilateral thalamus showed significant classification accuracies for Dataset 1, and the right S2 and the left insula showed significant classification accuracies for Dataset 2.


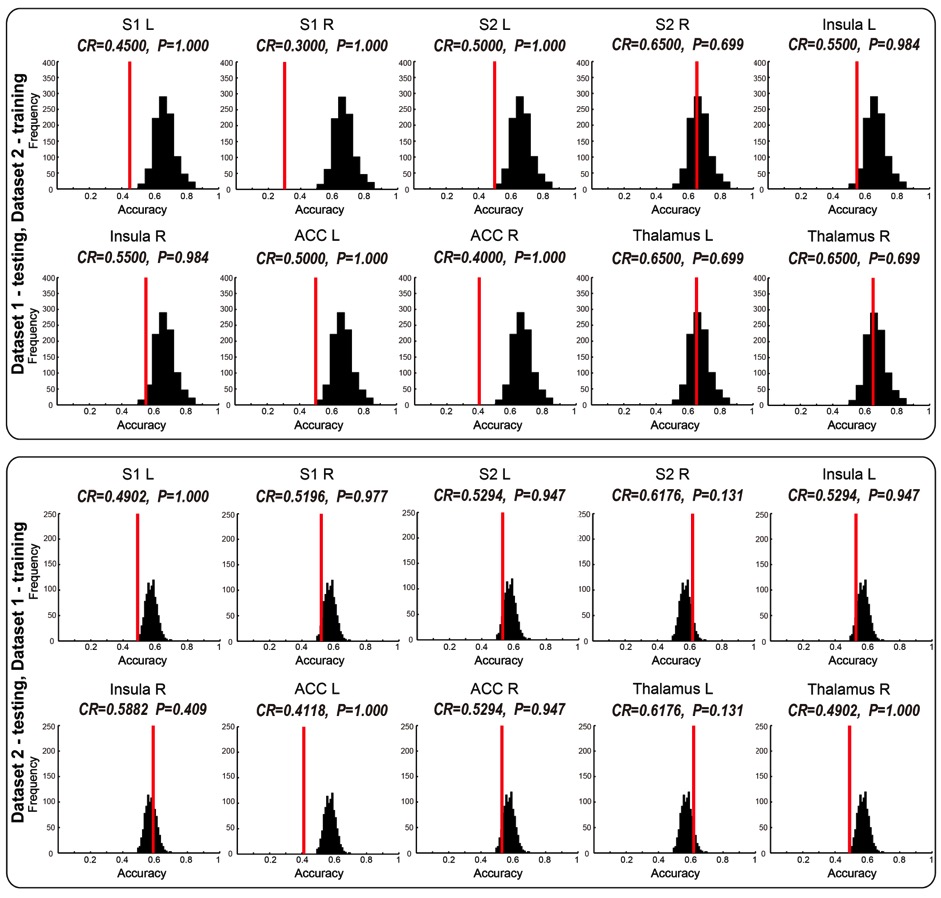


**Figure S13.** Pain vs. touch across-datasets classification accuracies obtained using normalised fMRI signals (upper panel: training using Dataset 2 and testing using Dataset 1; lower panel: training using Dataset 1 and testing using Dataset 2). Classification accuracies (correct rate, CR) are indicated by red vertical lines. The statistical significance was determined by 1,000 permutations and the resulting p-value was corrected for the 10 ROIs examined (FWE corrected), as follows. We took the maximal accuracy of the 10 ROIs for each permutation and then built a null distribution using the 1,000 maximal accuracies (i.e., the black bell shapes). None of the ROIs showed significant accuracies for across-datasets classifications.


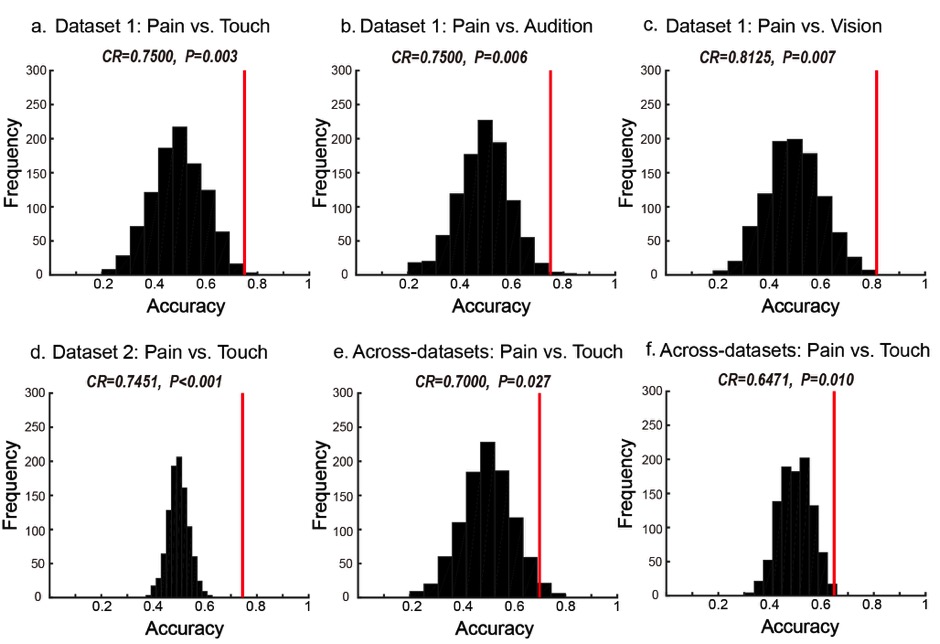


**Figure S14.** Classification accuracies (correct rates, CR) obtained using the normalized GLM beta maps of the “pain matrix” for the ‘pain vs. touch’ classification task. Panels **a-c**: classifications within Dataset 1. Panel **d**: classification within Dataset 2. Panels **e-f**: classifications across the two datasets: in panel **e** the classifier was trained using Dataset 2 and tested on Dataset 1; in panel **f** the classifier was trained using Dataset 1 and tested on Dataset 2. Classification accuracies are indicated by red vertical lines and corresponding null distributions (obtained from 1,000 permutations) are indicated by black bell shapes. P-values were calculated as the proportion of how many (out of 1,000) permutations generated accuracy greater than or equal to the actual classification accuracy.


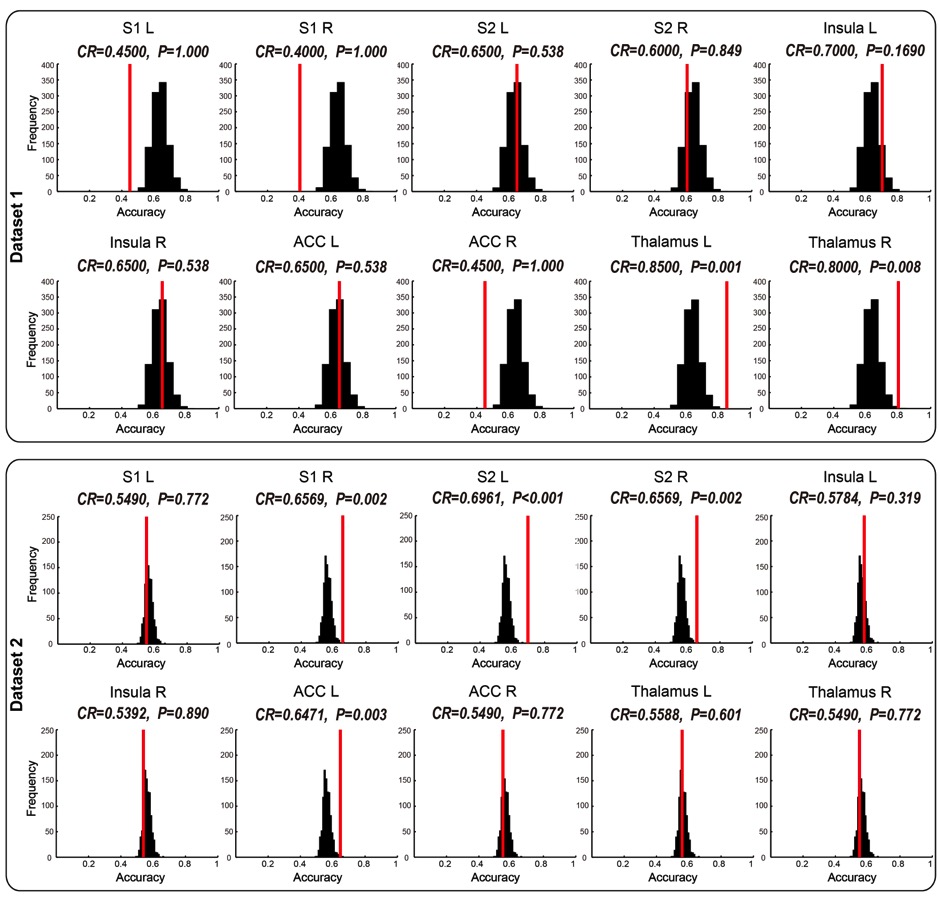


**Figure S15.** Pain vs. touch within-dataset classification accuracies obtained using normalised beta maps (upper panel: within Dataset 1; lower panel: within Dataset 2). Classification accuracies (correct rate, CR) are indicated by red vertical lines. The statistical significance was determined by 1,000 permutations and the resulting p-value was corrected for the 10 ROIs examined (FWE corrected), as follows. We took the maximal accuracy of the 10 ROIs for each permutation and then built a null distribution using the 1,000 maximal accuracies (i.e., the black bell shapes). The bilateral thalamus showed significant accuracies for Dataset 1, and the right S1, the bilateral S2 and the left ACC showed significant accuracies for Dataset 2.


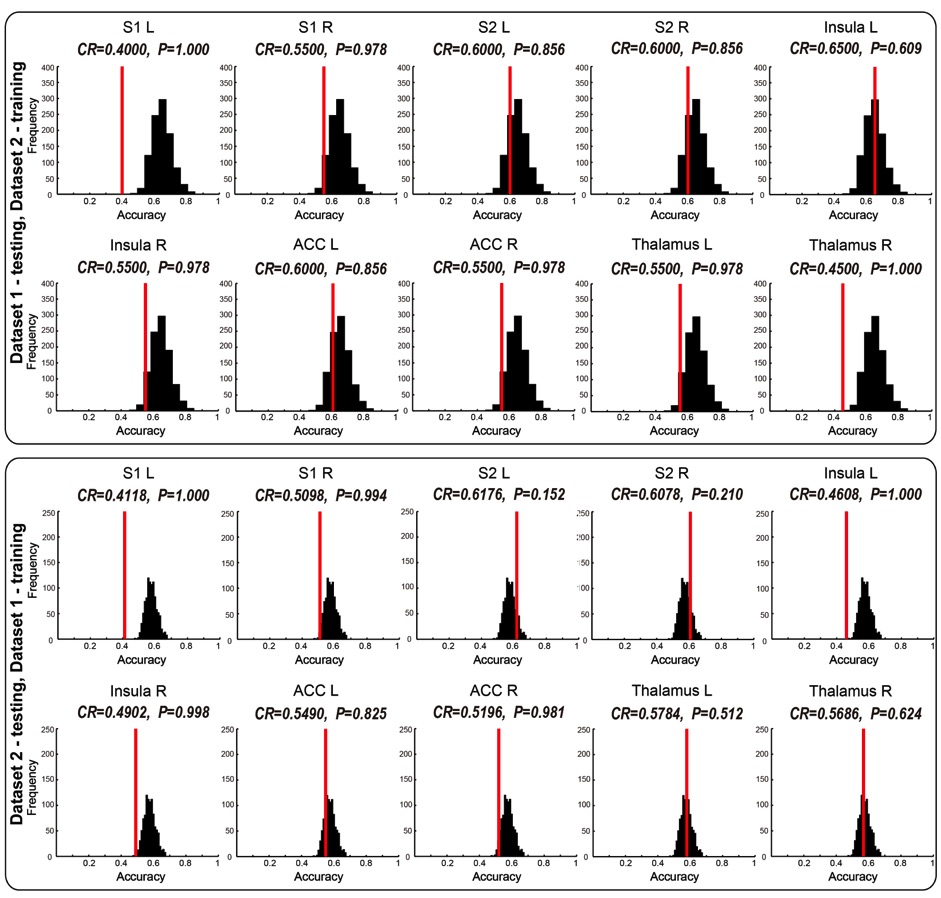


**Figure S16.** Pain vs. touch across-datasets classification accuracies obtained using normalised beta maps (upper panel: training using Dataset 2 and testing using Dataset 1; lower panel: training using Dataset 1 and testing using Dataset 2). Classification accuracies (correct rate, CR) are indicated by red vertical lines. The statistical significance was determined by 1,000 permutations and the resulting p-value was corrected for the 10 ROIs examined (FWE corrected), as follows. We took the maximal accuracy of the 10 ROIs for each permutation and then built a null distribution using the 1,000 maximal accuracies (i.e., the black bell shapes). None of the ROIs showed significant accuracies for across-datasets classifications.


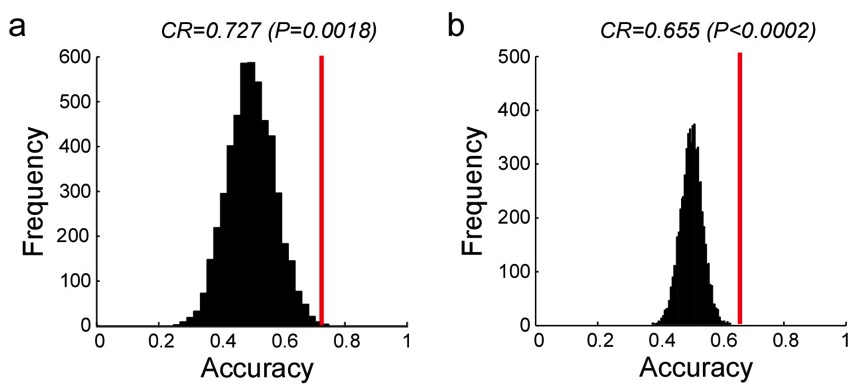


**Figure S17.** Classification accuracies (correct rates, CR) of ‘high vs. low intensity/saliency’ classification task obtained from non-normalized data, along with the corresponding null distributions, using Dataset 1 (**a**) and Dataset 2 (**b**). Classification accuracies are indicated by red vertical lines and corresponding null distributions (obtained from 5,000 permutations) are indicated by black bell shapes. P-values were calculated as the proportion of how many (out of 5,000) permutations generated accuracy greater than or equal to the actual classification accuracy.


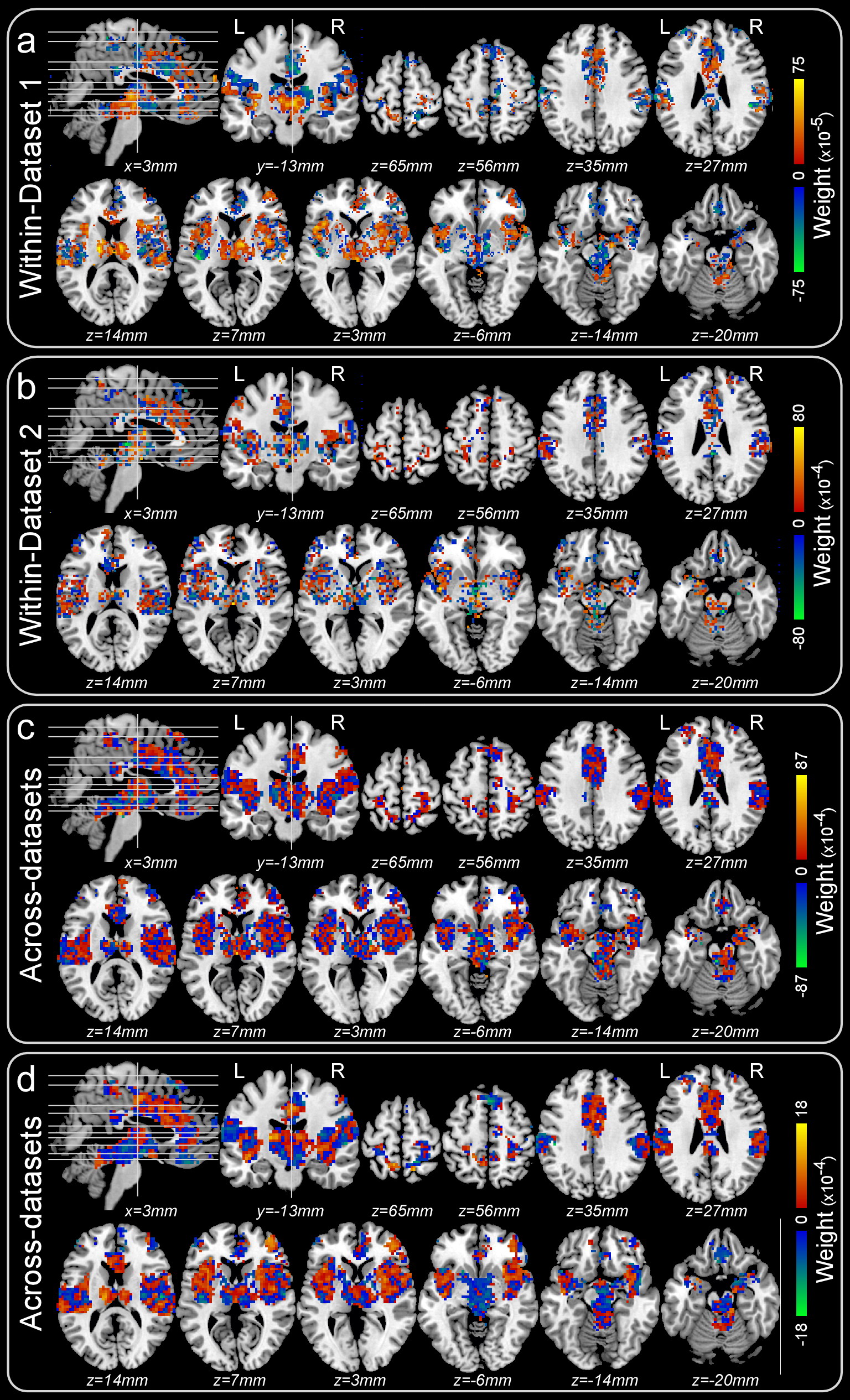


**Figure S18.** Sensitivity maps of ‘high vs. low intensity/saliency’ classification, within-dataset and across-datasets. Panel **a**: within Dataset 1 classification; panel **b**: within Dataset 2 classification; panel **c**: across-datasets classification, training using Dataset 2 and testing using Dataset 1; panel **d**: across-datasets classification, training using Dataset 1 and testing using Dataset 2.


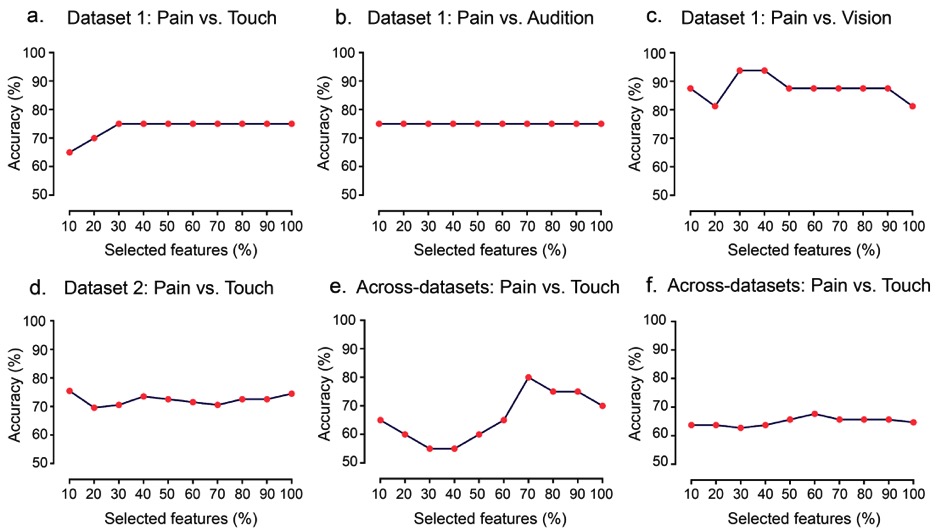


**Figure S19.** Classification accuracies as a function of the percentage of selected data features. Panel **a**: ‘pain vs. touch’ classification in Dataset 1. Panel **b**: ‘pain vs. audition’ classification in Dataset 1. Panel **c**: ‘pain vs. vision’ classification in Dataset 1. Panel **d**: ‘pain vs. touch’ classification in Dataset 2. Panel **e**: ‘pain vs. touch’ classification across datasets – with Dataset 2 used for training and Dataset 1 used for testing. Panel **f**: ‘pain vs. touch’ classification across datasets – with Dataset 1 used for training and Dataset 2 used for testing.

**Reference**

Chikazoe J, Lee DH, Kriegeskorte N, Anderson AK. 2014. Population coding of affect across stimuli, modalities and individuals. Nat Neurosci. 17:1114-1122.

Dienes Z, Coulton S, Heather N. 2018. Using Bayes factors to evaluate evidence for no effect: examples from the SIPS project. Addiction. 113:240-246.

Kim MJ, Mattek AM, Bennett RH, Solomon KM, Shin J, Whalen PJ. 2017. Human Amygdala Tracks a Feature-Based Valence Signal Embedded within the Facial Expression of Surprise. J Neurosci. 37:9510-9518.

Morey RD, Romeijn J-W, Rouder JN. 2016. The philosophy of Bayes factors and the quantification of statistical evidence. Journal of Mathematical Psychology. 72:6-18.

Nummenmaa L, Saarimaki H, Glerean E, Gotsopoulos A, Jaaskelainen IP, Hari R, Sams M. 2014. Emotional speech synchronizes brains across listeners and engages large-scale dynamic brain networks. Neuroimage. 102 Pt 2:498-509.

Rouder JN, Speckman PL, Sun D, Morey RD, Iverson G. 2009. Bayesian t tests for accepting and rejecting the null hypothesis. Psychon Bull Rev. 16:225-237.

Vigliocco G, Kousta ST, Della Rosa PA, Vinson DP, Tettamanti M, Devlin JT, Cappa SF. 2014. The neural representation of abstract words: the role of emotion. Cereb Cortex. 24:1767-1777.
